# Magnetic anomaly inversion through the novel barnacles mating optimization algorithm

**DOI:** 10.1038/s41598-022-26265-0

**Published:** 2022-12-30

**Authors:** Hanbing Ai, Khalid S. Essa, Yunus Levent Ekinci, Çağlayan Balkaya, Hongxing Li, Yves Géraud

**Affiliations:** 1 Laboratory for Fundamental Science on Radioactive Geology and Exploration Technology, East China University of Technology, No. 418 Guanglan Avenue, Nanchang, 330013 Jiangxi China; 2 Geophysics Department, Faculty of Science, Cairo University, P.O. 12613, Giza, Egypt; 3 Department of Art History, Bitlis Eren University, 13100 Bitlis, Türkiye; 4 Department of Geophysical Engineering, Süleyman Demirel University, 32260 Isparta, Türkiye; 5 GeoRessources Laboratory, University of Lorraine, 54500 Nancy, France

**Keywords:** Environmental sciences, Solid Earth sciences

## Abstract

Dealing with the ill-posed and non-unique nature of the non-linear geophysical inverse problem via local optimizers requires the use of some regularization methods, constraints, and prior information about the Earth's complex interior. Another difficulty is that the success of local search algorithms depends on a well-designed initial model located close to the parameter set providing the global minimum. On the other hand, global optimization and metaheuristic algorithms that have the ability to scan almost the entire model space do not need an assertive initial model. Thus, these approaches are increasingly incorporated into parameter estimation studies and are also gaining more popularity in the geophysical community. In this study we present the Barnacles Mating Optimizer (BMO), a recently proposed global optimizer motivated by the special mating behavior of barnacles, to interpret magnetic anomalies. This is the first example in the literature of BMO application to a geophysical inverse problem. After performing modal analyses and parameter tuning processes, BMO has been tested on simulated magnetic anomalies generated from hypothetical models and subsequently applied to three real anomalies that are chromite deposit, uranium deposit and Mesozoic dike. A second moving average (SMA) scheme to eliminate regional anomalies from observed anomalies has been examined and certified. Post-inversion uncertainty assessment analyses have been also implemented to understand the reliability of the solutions achieved. Moreover, BMO’s solutions for convergence rate, stability, robustness and accuracy have been compared with the solutions of the commonly used standard Particle Swarm Optimization (sPSO) algorithm. The results have shown that the BMO algorithm can scan the model parameter space more extensively without affecting its ability to consistently approach the unique global minimum in this presented inverse problem. We, therefore, recommend the use of competitive BMO in model parameter estimation studies performed with other geophysical methods.

## Introduction

The magnetic method has so far been used in a wide range of investigations at various sites^1–11^. The most important task of the magnetic method is to explore subsurface geology using minor changes in the geomagnetic field caused by magnetized masses. As with other geophysical methods, magnetic anomalies are assessed by making some assumptions and generalizations. Magnetized masses are commonly analyzed using some simple-shaped source structures^12,13^. However, these geometrically idealized source structures cannot be considered geologically perfect, but are used in the investigation of the magnetic anomalies to simplify modelling and interpretation procedures^14–17^. In order to better interpret magnetized causative structures, many computational approaches have been proposed, such as matching curve^18,19^, Fourier and Hilbert Transforms^20^, characteristics points and distances^21^, Least-squares approximations^22–24^, Euler Deconvolution^25^, simplex algorithm^26^, correlation techniques^27^, variance analyses^28^, derivative-based and moving average-based algorithms^29,30^, spectral analysis techniques^31^, and local wave number techniques^32^. In addition to these methodologies, the most frequently used tool for interpreting magnetic anomalies is the inversion approach. In geophysics, reconstructing the appropriate subsurface model using a set of observations is known as inversion or inverse problem. Owing to the well-acknowledged ill-posed and non-unique nature of magnetic inverse problems, estimations of the model parameters of buried magnetized sources can be achieved through some challenging processes^33–37^. Local and global search algorithms can be applied for the inversion procedure^21,38^. Derivative-based local search algorithms using various regularization procedures have been routinely used for this task. Despite their very fast convergence characteristics, they have some serious disadvantages. These algorithms require *prior* information depending on geological conditions. Moreover, their success largely depends on the choice of initial guess and therefore they cannot scan the entire model space. They typically tend to reach a minimum in the vicinity of the initial model parameter set. Thus, these optimizers may reach any local minimum instead of the global minimum which is the deepest valley in the objective function surface topography. On the other hand, derivative-free global optimization and metaheuristic algorithms use a random walk search to reach the minimum in the given model parameter space. They can effectively scan the entire model space and therefore do not require a well-designed initial guess. Additionally, they have the ability of escaping local minima in the complex nature of the error function topography. Because of these advantages, the use of such nature-inspired algorithms in geophysical inverse problems has become very popular over the last decade. In fact, the first applications of these intelligence algorithms date back to the years before 1940, which is called the pre-theoretical period when the studies were informal^39^. The period between 1940 and 1980 is the early period, and these algorithms were formally studied during this period. In the method-centric period which is between 1980 and 2000 metaheuristic studies increased dramatically and numerous different approaches were introduced as an alternative tool to classical optimization algorithms^40^. The period from the 2000s to the present is called the framework-centric period, and the idea of describing computational intelligence algorithms as frameworks rather than methods has increased^39^.

Standard Particle Swarm Optimization (sPSO) and Genetic Algorithm (GA) are commonly used in geophysical inversion^41^. Some comparative studies have verified that sPSO outperforms GA in terms of accurateness and better convergence characteristics for various problems^42–45^. However, it should be noted that there is no optimal metaheuristic algorithm for solving all types of inverse problems. Therefore, new problem-specific global optimization algorithms are still being developed. Accordingly, adaptations of new nature-inspired derivative-free algorithms for the inversion of magnetic anomalies have taken their place in the literature. In most cases, the outputs obtained were not compared with the results of another metaheuristic algorithm. Besides, in most studies, possible uncertainties in the model parameters obtained were not investigated. However, it is a well-known fact that global optimization and metaheuristic algorithms allow to perform uncertainty analysis which is an essential step in understanding the reliability of the solutions obtained. The difficulty of the inverse problem is increased when multiple-source structures are used to represent the resulting magnetic anomalies. Therefore, some of the anomaly peaks are not used in most studies, and the number of source structures is reduced and attempts are made to find a solution. In addition, it is an important deficiency that the possible regional magnetic anomaly effect is mostly ignored. Table 1 lists information about magnetic anomaly inversion studies carried out in recent years with global optimization algorithms and the presented study. It is clear that comparative tests, uncertainty analyses and regional effect investigations were not performed together in previous studies. In this study, we present a novel bio-inspired algorithm called Barnacles Mating Optimizer (BMO) for the inversion of magnetic anomalies. This optimization algorithm imitates the unique mating character of barnacles. Three theoretical cases with different scenarios were used to understand the proficiency of the proposed algorithm. Besides, three real data sets, including a chromite ore anomaly (India), an uranium ore anomaly (India) and an intrusive Mesozoic dyke anomaly (Brazil) were taken into consideration. Prior to the inversion experiments, we performed some modal analyses by mapping the surface topographies of the objective function for the model parameter pairs. Thuswise the resolvabilities of model parameters and their effects on objective function were explored. Following the previous works^9,46^ we also investigated the regional background effect through the second moving average (SMA) technique. Consistency and credibility of the solutions obtained were assessed by performing some uncertainty appraisal analyses. Finally, the performances of the BMO and sPSO algorithms in our case were compared.

**Table 1:** Magnetic anomaly inversion studies carried out in recent years with global optimization algorithms and the presented study.

Algorithm	Inspiration	Compared with	Uncertainty analyses	Regional analyses	Magnetic anomaly
Ant-Colony Optimization (ACO)^47^	Strategies of ant colonies to find the shortest path	PSO	No	No	Six sources
Simulated Annealing Optimization (SAO)^34^	Metal annealing process	None	Yes	No	Single source
Genetic Algorithm (GA)^48^	Evolutionary procedures	None	No	No	Single source
Differential Evolution Algorithm (DEA)^49^	Evolutionary procedures	None	No	No	Two sources
Grey Wolf Optimization (GWO)^50^	Hierarchical behaviors of grey wolves for hunting	PSO	No	No	Four sources
Particle Swarm Optimization (PSO)^51^	Behaviors of foraging birds and fish	None	No	No	Two sources
Genetic Price Algorithm (GPA)^52^	Genetic random search procedure	None	No	No	Three sources
Whale Optimization (WO)^53^	Strategies of humpback whales seeking prey	None	No	No	Single source
Differential Search Algorithm (DSA)^54^	Superorganism migrations	None	Yes	No	Two sources
Adaptive Differential Evolution (ADE)^55^	Evolutionary procedures	None	No	No	Two sources
Bat Algorithm Optimization (BAO)^56^	Echolocation behaviors of microbats	None	No	No	Two sources
Manta-Ray Foraging Optimization (MFO)^57^	Intelligent behaviors of manta-rays	PSO, SA, GA	No	No	Two sources
Social Spider Optimization (SSO)^58^	Foraging strategies of social spiders	None	Yes	No	Two sources
Hybrid PSO-GA^59^	Behaviors of foraging birds and fish with genetic operators	None	No	No	Single source
Barnacles Mating Optimizer (BMO)	Mating behavior of barnacles	PSO	Yes	Yes	Four sources

## Methodology

### Forward modeling

**Figure 1:**
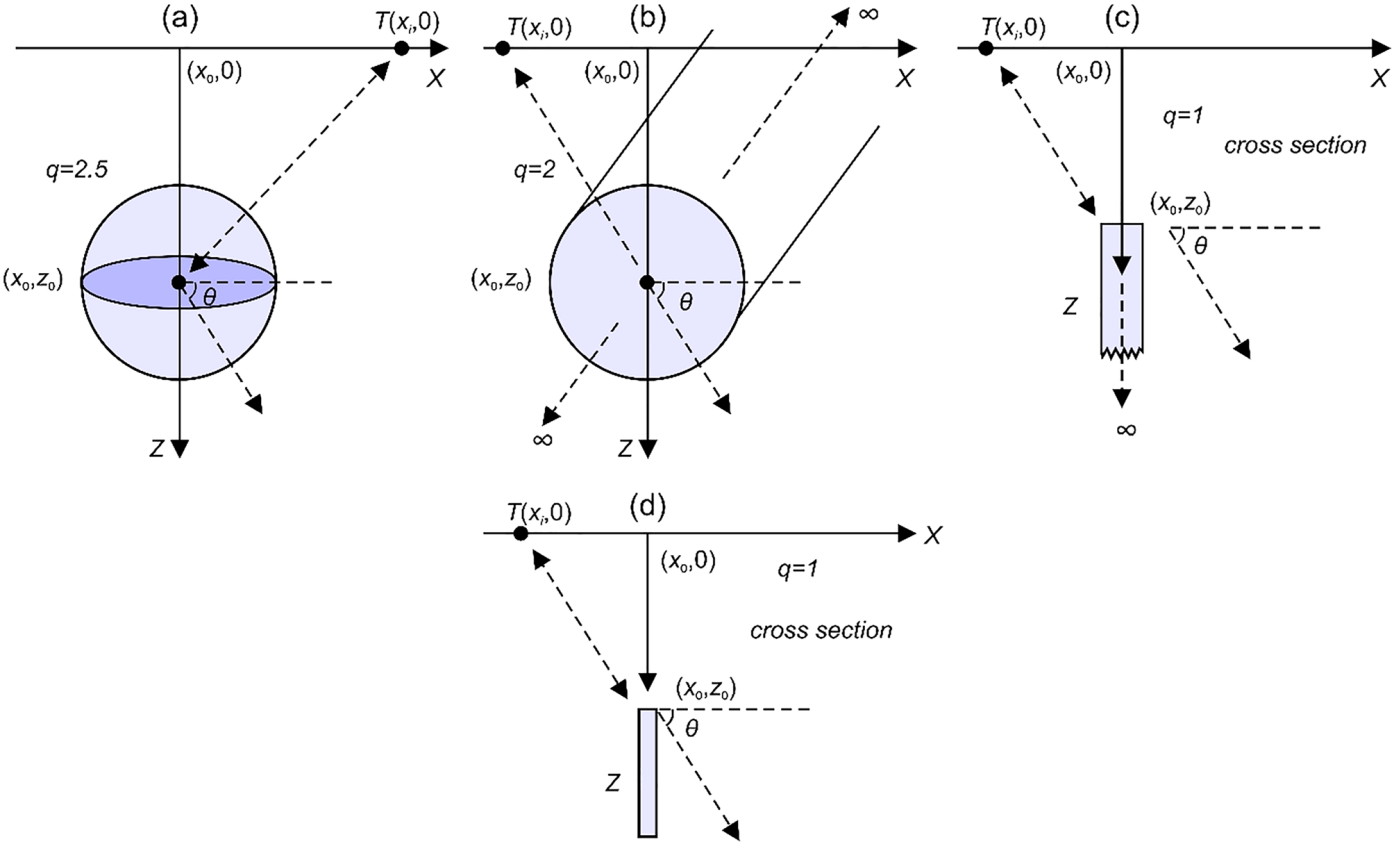
Illustrations of geometries and parameters of (**a**) a sphere, (**b**) an infinitely long horizontal cylinder, (**c**) a thin dyke, and (**d**) a thin sheet.

The magnetic anomaly equations for some idealized sources (Fig. 1) such as a sphere^60,61^, an infinitely long horizontal cylinder^62^, a thin dyke^23,63,64^ and a thin sheet^63^ are given below, respectively.

1 $$T(x_{i} ) = K \times z_{0}^{3} \left\{ {\frac{{\left[ {2z_{0}^{2} - \left( {x_{i} { - }x_{0} } \right)^{2} } \right] \times \sin \theta + 3z_{0} \left( {x_{i} { - }x_{0} } \right) \times \cos \theta }}{{\left[ {\left( {x_{i} { - }x_{0} } \right)^{2} + z_{0}^{2} } \right]^{q} }}} \right\},\;i = 1,\;2,\;3...\;{\text{M,}}$$

2 $$T(x_{i} ) = K\left\{ {\frac{{\left[ {z_{0}^{2} - \left( {x_{i} { - }x_{0} } \right)^{2} } \right] \times \cos \theta + 2z_{0} \left( {x_{i} { - }x_{0} } \right) \times \sin \theta }}{{\left[ {\left( {x_{i} { - }x_{0} } \right)^{2} + z_{0}^{2} } \right]^{q} }}} \right\},\;i = 1,\;2,\;3...\;{\text{M,}}$$

3 $$T(x_{i} ) = K \times z_{0} \left\{ {\frac{{\left( {x_{i} { - }x_{0} } \right) \times \sin \theta + z_{0} \times \cos \theta }}{{\left[ {\left( {x_{i} { - }x_{0} } \right)^{2} + z_{0}^{2} } \right]^{q} }}} \right\},\;i = 1,\;2,\;3...\;{\text{M,}}$$

4 $$T(x_{i} ) = K\left\{ {\frac{{z_{0} \times \cos \theta - \left( {x_{i} { - }x_{0} } \right) \times \sin \theta }}{{\left[ {\left( {x_{i} { - }x_{0} } \right)^{2} + z_{0}^{2} } \right]^{q} }}} \right\},\;i = 1,\;2,\;3...\;{\text{M}}{.}$$

In the definitions given above, *T* is the total field magnetic anomaly, *x*_*i*_ is the observation point on a profile, *z*_0_ (m) represents the depth to the center of the buried magnetic source (spheres and cylinders) and the top of dykes and thin sheets, *θ* denotes the effective magnetization angle (degree), *q* represents the shape factor (dimensionless), *x*_0_ defines the origin of the anomaly or the horizontal center coordinate of the magnetic source (m), and *K* denotes the coefficient of amplitude (nT × m^2*q*−2^), which is related to the model shape. The shape factors 2.5, 2, 1, and 1 are used for spheres, horizontal cylinders, thin dykes, and thin sheets, respectively^12^. On the basis of principle of superposition, composite magnetic anomalies of multiple-source are calculated easily using the following definition^12^.

5 $$T_{p} (x_{i} ) = \sum\limits_{j = 1}^{P} {T_{j} (x_{i} )} ,$$

where *T*_*j*_ and *P* are the magnetic anomaly of *j*-th source and the number of sources, respectively.

### Undesired regional effect and local interruption

It is well-acknowledged that, due to the heterogeneity of the Earth’s interior, the measured residual magnetic anomaly of a certain shallow source is mostly corrupted by undesired regional effects and some local interferences. The mathematical definitions of these effects are given as follows^9^:

6 $$T_{gen} (x_{i} ) = T_{p} (x_{i} ){ + }T_{re} (x_{i} ){ + }T_{local} (x_{i} ),$$

$$T_{re} (x_{i} ) = \sum\limits_{k = 1}^{3} {{\text{c}}_{k} x_{i}^{k} + } {\text{c}}_{0} ,$$

where *T*_*gen*_(*x*_*i*_) denotes the composite anomaly, *T*_*re*_(*x*_*i*_) is the undesired regional effect (c_0_–c_3_ are predefined constants), and *T*_*local*_(*x*_*i*_) represents the local perturbations originated from interfering sources.

### SMA method

A residual magnetic response of a shallow source can be calculated by removing the regional effect from the observed data set. A recent study^9^ reported the efficiency of the SMA method to achieve this goal using the following definition:

7 $$R_{2} (x_{i} ) = \frac{{6T_{gen} (x_{i} ) - 4T_{gen} (x_{i} + s) - 4T_{gen} (x_{i} - s) + T_{gen} (x_{i} + 2s) + T_{gen} (x_{i} - 2s)}}{4},$$

where *R*_2_(*x*) is the approximated residual magnetic anomaly, and *s* (m) denotes the window length. Typically, in order to provide a reasonable estimate of the true model parameters we can simply use the mean output produced by delimiting the SMA magnetic anomalies, which is controlled by s values (window length).

### BMO

BMO is a novel algorithm^65^, which is used to simulate the special mating behaviors of barnacles for the optimization of some engineering problems. Barnacles are mostly hermaphrodites and live in shallow and tidal waters. These fantastic individuals can attach themselves temporarily to substratum or symbionts in the water. For this purpose, they mostly use whales, sea snakes or any other crustaceans. In order to survive, barnacle mating groups surround neighbors and potential competitors within reach of their penises.

The reported^65^ satisfactory results of BMO in 23 challenging benchmark functions and power system analyses motivated us to apply it to nonlinear geophysical problems. Applications with many different scenarios are presented to show how the proposed algorithm can reach the global optimum without suffering from the local optima entrapments and without being affected by the ill-posed nature of the magnetic inverse problem. The steps of the algorithm are given in brief as follows.

#### Initialization

In BMO, it is assumed that barnacles are the solutions^65^. Thus, in the inverse problem presented here barnacles can be treated as the model parameters (*K*, *θ*, *x*0, *z*0, *q*). The matrix of population **X** is defined as follows:

8 $${\mathbf{X}} = \left( {\begin{array}{*{20}c} {X_{1}^{1} } & \ldots & {X_{1}^{dim} } \\ \vdots & \ddots & \vdots \\ {X_{N}^{1} } & \cdots & {X_{N}^{dim} } \\ \end{array} } \right),\;X_{j}^{i} \in [lb_{i} ,\;ub_{i} ],$$

where *dim* is the number of control variables (*dim* = 5 in this study) and *N* denotes the number of barnacles (population size). The $X_{j}^{i}$ (the *i*-th control variable of the *j*-th barnacle. Here, *i* = 1, 2, 3... *dim*, *j* = 1, 2, 3... *N*) given above is subject to the upper and lower bounds [*lb*_*i*_, *ub*_*i*_]. The appraisal of **X** is terminated primarily, and the sorting process based on the obtained fitness values is carried out to detect the best solution (*K*, *θ*, *x*_0_, *z*_0_, *q*) at the top of **X**.

#### Selection process

BMO uses a different system compared to other evolution-based systems such as Genetic algorithm^66^, Differential Evolution^67^ etc. As the selection of two barnacles is relied on the length of their penises (*pl*) a simple case can be presented^65^ to illustrate this special mating behavior assuming that the best solution is located at the top of **X** at a particular iteration and the maximum penis length of barnacles *pl* = 7. Therefore, barnacle #1 is only able to mate with one of barnacles #2 and #7 (selected barnacles are located within *pl*). Hence, the following simple mathematical forms to achieve the selection process is proposed^65^:

9 $$\begin{array}{*{20}l} { barnacle_{d} = {\text{randperm}} \left( N \right)} \hfill \\ { barnacle_{m} = {\text{randperm}} \left( N \right),} \hfill \\ \end{array}$$

where randperm(*N*) is a function that returns a row vector that contains a random permutation of the integers from 1 to *N* inclusive. The *barnacle*_*d*_ and *barnacle*_*m*_ are the parents to be mated and they should locate within *pl*. However, if one of them does not fulfill this requirement, the normal mating process is therefore postponed. The next generation is updated using the sperm casting process instead.

#### Reproduction

BMO’s reproduction progression differs from other evolution-based algorithms. There is no explicit formula for developing the reproduction of barnacles, so BMO produces offspring based on the principle of the Hardy–Weinberg^68,69^. The following expression is given for this procedure:

10 $$X_{j\_new}^{i} = pX_{{barnacle_{d} }}^{i} + qX_{{barnacle_{m} }}^{i} ,$$

where *p* represents random numbers in the range of [0, 1], *q* = (1 − *p*), $X_{{barnacle_{d} }}^{i}$ and $X_{{barnacle_{m} }}^{i}$ are selected parents using Eq. (9). *p* and *q* denote the percentage of characteristics of the mating objects implanted in the next generation. The offspring, therefore, inherits the behaviors depending on the probability of the random number in the range of [0, 1].

**Figure 2:**
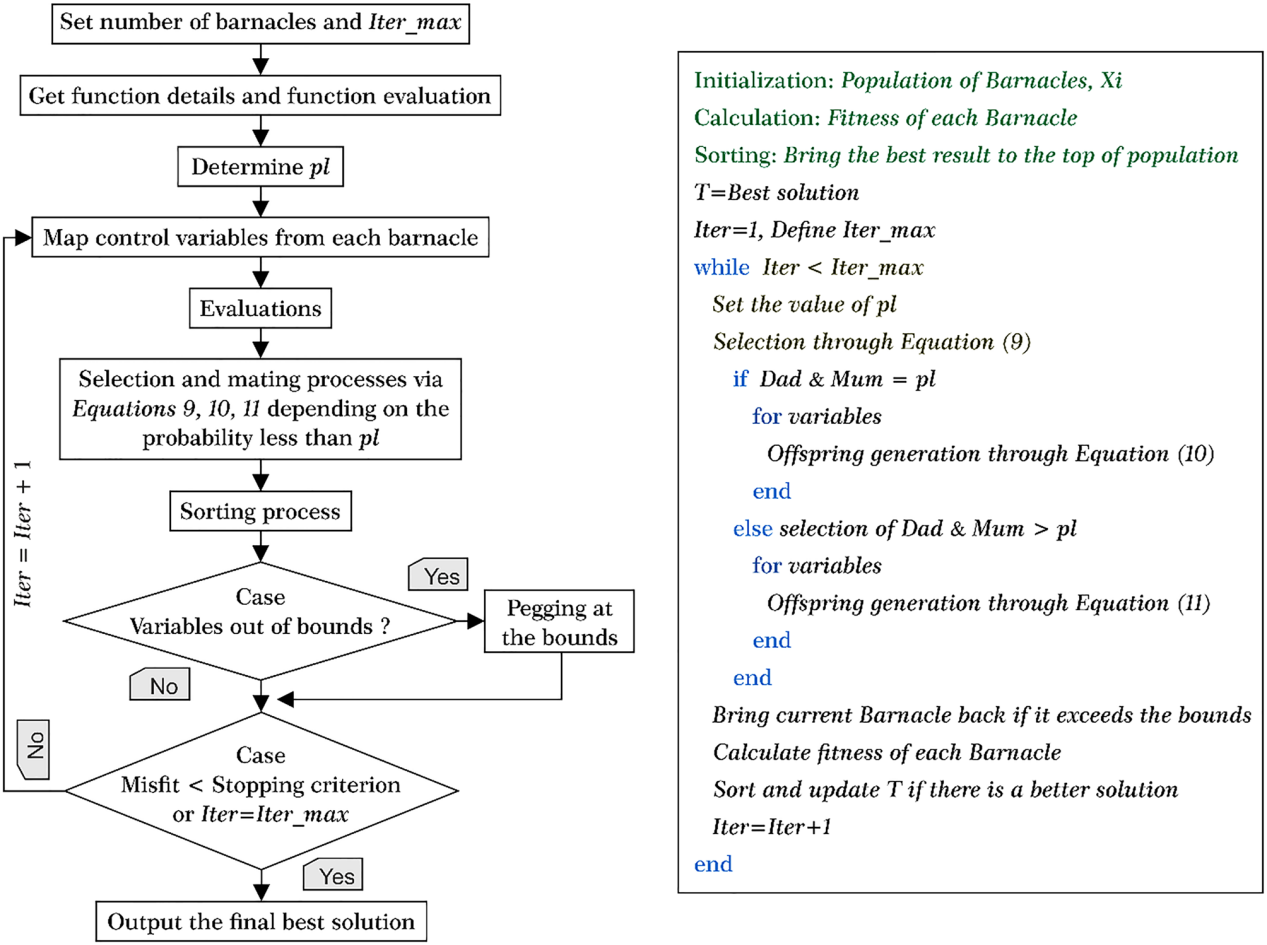
Workflow and pseudocode of the BMO (modified from the original study^65^).

In the BMO algorithm *pl* has an important impact on the exploitation (normal mating process) and exploration stages. The exploitation process occurs if the selected barnacle to be mated is within the penis length of the male barnacle. If not, the sperm cast is implemented and considered as an exploration process of BMO as given below:

11 $$X_{j\_new}^{i} = rand() \times X_{{barnacle_{m} }}^{i} ,$$

where rand() denotes random numbers between [0, 1]. Along with Eq. (11), the new offspring is formed by the female barnacle because the female barnacle retrieves the sperm from the water left by the other barnacles. As can be seen from the brief introduction to BMO, *pl* is the algorithm-based control parameter, which needs to be selected before employing BMO. The tuning process of *pl* will be discussed later. Figure 2 describes the basic workflow and pseudocode of BMO algorithm.

### Objective function and stopping criterion

Basically, the BMO algorithm uses model parameters as search agents, and then quantitatively simulated specific matching behaviors guide these search agents to converge iteratively. Finally, if the *root-mean-square-error* (*RMSE*) is minimized to a predefined small value or the iteration number reaches the predefined maximum number (*Iter_max*), the iteration cycle is terminated. Subsequently, the model parameter set (*K*, *θ*, *x*_0_, *z*_0_, *q*) obtained at the last iteration is considered to be the optimal solution. The definition of *RMSE* used in our case is given as follows:

12 $$RMSE = \sqrt {\sum\limits_{i = 1}^{\text{M}} {\frac{{\left[ {T_{obs} (x_{i} ) - T_{cal} (x_{i} )} \right]^{2} }}{{\text{M}}}} } ,$$

where *T*_*obs*_ is the observed data, *T*_*cal*_ denotes the data calculated from the model solution, *x*_*i*_ is the *i*-th data measuring point along the profile aforementioned, and M represents the number of observed points. The relative error between the true and estimated parameter sets is determined by:

13 $$Relative\;error = \frac{{{\text{abs}} \left( {S_{true} - S_{est} } \right)}}{{{\text{abs}} (S_{est} )}},$$

where abs() is an absolute function, *S*_*true*_ and *S*_*pre*_ denote the true and the estimated model parameter set.

## Synthetic applications

### Model analysis of the objective function

The ill-posedness characteristics of the geophysical inverse problem makes the model parameter estimation process suspect and error-prone^70,71^. Hence, pre- and post-inversion uncertainty appraisal studies play a crucial role in the detection of the ambiguities and consistencies in the obtained source parameters^36^. Commonly used pre-inversion analyses in geophysical inverse problems is the study of the modal type of the objective function used. These analyzes allow us to make a preliminary assessment of the mathematical solvability of the inverse problem of interest. We used the following definition as the objective function in the applications.

14 $$RMSE = \sqrt {\sum\limits_{i = 1}^{\text{M}} {\frac{{\left[ {T_{p = 4} (x_{i} ) - T_{cal} (x_{i} )} \right]^{2} }}{{\text{M}}}} } ,$$

where $T_{{p{ = }4}} (x_{i} )$ is the simulated magnetic anomaly generated by a multiple-source model, where its true parameter set (*K*, *θ*, *x*_0_, *z*_0_, *q*) is presented in Table 2. The following definition were used to generate the synthetic data set:

15 $$\begin{aligned} T_{p = 4} (x_{i} ) & = 60 \times 8^{3} \left\{ {\frac{{\left[ {2 \times 8^{2} - \left( {x_{i} - 30} \right)^{2} } \right] \times \sin 60^\circ + 3 \times 8 \times \left( {x_{i} - 30} \right) \times \cos 60^\circ }}{{\left[ {\left( {x_{i} - 30} \right)^{2} + 8^{2} } \right]^{5/2} }}} \right\} \\ & \quad + 2000\left\{ {\frac{{\left[ {5^{2} - \left( {x_{i} + 25} \right)^{2} } \right] \times \cos 30^\circ + 2 \times 5 \times \left( {x_{i} + 25} \right) \times \sin 30^\circ }}{{\left[ {\left( {x_{i} + 25} \right)^{2} + 5^{2} } \right]^{2} }}} \right\} \\ & \quad { + }50 \times 20 \times \left\{ {\frac{{\left( {x_{i} { - }120} \right) \times \sin 10^\circ + 20 \times \cos 10^\circ }}{{\left[ {\left( {x_{i} { - }120} \right)^{2} + 20^{2} } \right]}}} \right\}{ + }800\left\{ {\frac{{12 \times \cos 50^\circ - \left( {x_{i} + 100} \right) \times \sin 50^\circ }}{{\left[ {\left( {x_{i} + 100} \right)^{2} + 12^{2} } \right]}}} \right\}. \\ \end{aligned}$$

**Table 2:** True model parameters of the multi-source model. Parameter variation ranges are also given.

	Spherical source	Horizontally-placed infinite cylindrical body	Semi-infinite thin dyke	Semi-infinite thin sheet
**True parameters**				
*K* (nT × m^2*q*−2^)	60	2000	50	800
*θ* (degree)	60	30	10	50
*x*_0_ (m)	30	− 25	120	− 100
*z*_0_ (m)	8	5	20	12
*q* (dimensionless)	2.5	2	1	1
**Variation range**				
	30 ~ 90	1000 ~ 3000	25 ~ 75	400 ~ 1200
	30 ~ 90	15 ~ 45	5 ~ 15	25 ~ 75
	15 ~ 45	− 37.5 ~ − 12.5	60 ~ 180	− 150 ~ − 50
	4 ~ 12	2.5 ~ 7.5	10 ~ 30	6 ~ 18
	1.25 ~ 3.75	1 ~ 3	0.5 ~ 1.5	0.5 ~ 1.5

**Figure 3:**
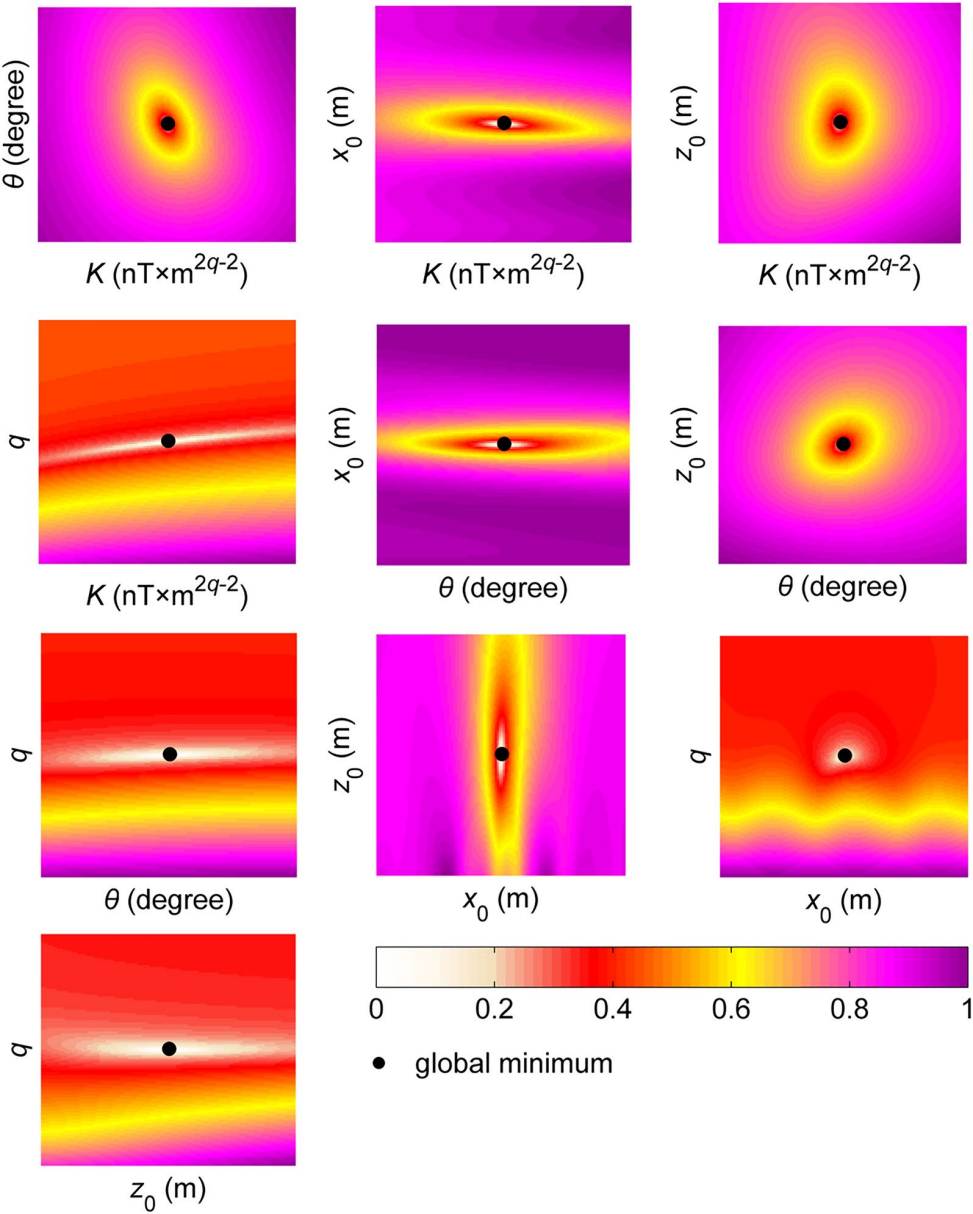
Modal analyses of the defined objective function for model parameter pairs.

Synthetic magnetic anomaly was generated along a 400 m long profile with 10 m data intervals. We considered a range of values that are half and twice (50% perturbations) the true model parameter values to produce a wide search space for the modal analyses. Figure 3 shows the distributions of the cost function. The middle points of these surface topography maps locate the true values of the source parameter pairs (the global minimum). The distribution maps of *θ* − *K*, *z*_0_ − *K* and *z*_0_ − *θ* pairs clearly revealed that the global minima values are enclosed with almost rounded contours and no local ones are situated, which indicates the uni-modal feature^72^. In short, the parameter pairs are uncorrelated and can most likely be estimated individually by executing an efficient inversion code. However, some elongated valleys and basins with different flat bottoms representing the lowest error function regions displaying the multi-modal^73^ characteristics were observed in other maps (*θ* − *x*_0_, *θ* − *q*, *z*_0_ − *q*, *q* − *x*_0_, *q* − *K*, *θ* − *K*, and *x*_0_ − *z*_0_ in Fig. 3), which make the problem complicated, decrease the resolvability characteristics of the source parameters, and increase the uncertainties in the solutions. This means that parameter pairs are correlated with each other. It is well-known fact that banana-shaped contours in objective function surface topographies around the global minimum make precise estimations almost impossible. In our case, we did not observe this kind of topographic features. Thus, model parameters of the non-linear inverse problem presented here can most likely be resolved. Based on these findings, it is clear that the objective function yields the composite modality^74^ since the uni-modality and multi-modality exist together regarding all model parameter pairs. Error surface topographies of the objective function used indicate the characteristics of non-linearity, high dimensionality and various shapes in paired model spaces, which means that estimations of the model parameters are challenging. However, these difficulties can be overcome with a powerful inversion algorithm which has sufficient ability to approach the global minimum as close as possible without compromising its robustness in order not to be captured by the local minima.

### Parameter tuning process

**Figure 4:**
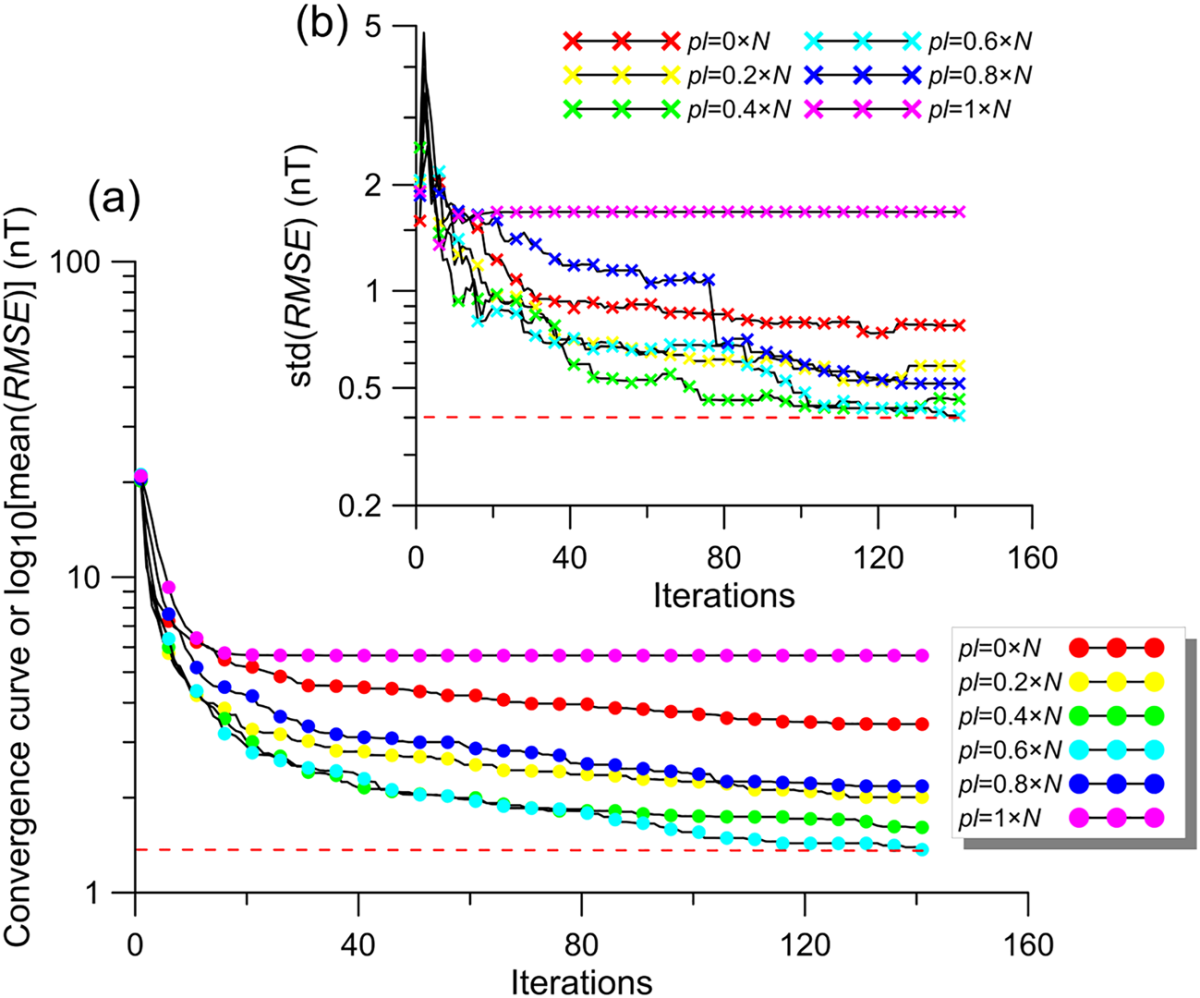
Analyzing the effect of various *pl* values. (**a**) Variation behavior of the mean *RMSE* values obtained via the increment of *pl*, (**b**) uncertainties of BMO via the variation of *pl*.

The efficiency of the global optimization algorithm largely depends on fine-tuned control parameters. However, the control parameters proposed by the developers for their own problems or reported ones in previous studies are commonly used for many types of optimization problems. It must be noted that there is no algorithm-based control parameter value that can be successful for all types of inverse problems. These optimal values may vary depending on the nature of inverse problems^49,70,75^. Hence, a vital step of applying a metaheuristic effectively is to appropriately determine the best user-defined control parameter value/s. As already mentioned, *pl* plays a key role in defining the exploitation (normal mating process) and exploration stages (sperm cast process) of BMO. To understand the effect of *pl* values on the solutions a composite noise-free magnetic anomaly due to a multiple-source model was inverted (model parameters are given in Table 2). The search space values used in modal analyses were considered. We used 30 independent runs to suppress the stochastic nature of the metaheuristics so that a more objective evaluation and estimation can be achieved. The optimization processes were performed using 140 iterations and *N* = 80. As the *pl* of the BMO varies successively (from 0 × *N* to 1 × *N*), the obtained mean *RMSE* values are displayed in Fig. 4a. Figure 4b demonstrates the variation of the standard deviations (stds) of the calculated *RMSE* values, indicating the uncertainty of inversion. The variations in the curves (Fig. 4a,b) obtained via various *pl* values clearly showed that an ill-defined *pl* value can reduce the performance of the BMO significantly in terms of affecting the solution correctness and the stableness of searching the true parameter values. Additionally, contrary to our expectations, both smaller (e.g., *pl* = 0 × *N*) and larger (e.g., *pl* = 1 × *N*) *pl* values caused insufficient performances. By this way, instead of making a random guess based on intuition, the importance of determining the optimum control parameter value/s for the problem of interest was revealed. By performing many trial-and-error tests, we determined that 0.65 is the optimum value for the *pl*.

### Inversion studies

We used three synthetic cases with different scenarios to investigate the performance of BMO. The first case discusses a residual magnetic anomaly originated from a multiple-source model given in Table 2. In the second case, we added a regional background effect using a 3rd order polynomial and a local anomaly caused by a near-surface interference (spherical source) to the first case anomaly. In the last case, the second case anomaly was contaminated with a certain degree of random noise (40%). The solutions obtained through the BMO algorithm were compared with those of the sPSO algorithm that uses the suggested^9,51^ control parameters. Besides, we investigated the performance of the SMA operator in reducing the effect of the regional background. All experiments were conducted on a Windows 10 operating system with Intel(R) Core (TM) i5-6300HQ CPU (2.30 GHz) and 3.8 GB of RAM.

#### Case 1

**Figure 5:**
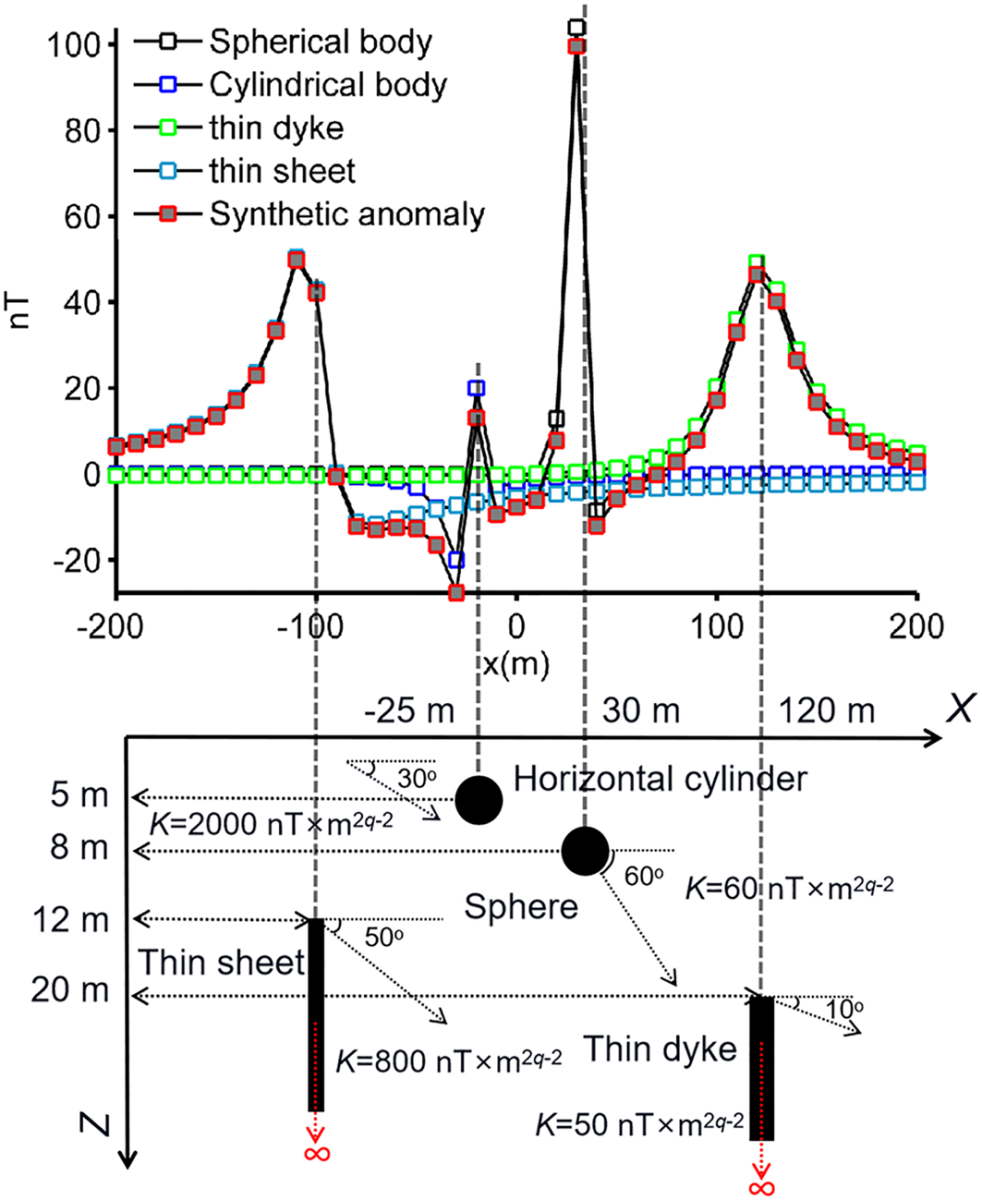
Simulated magnetic anomaly of multiple-source and the causative subsurface model.

**Figure 6:**
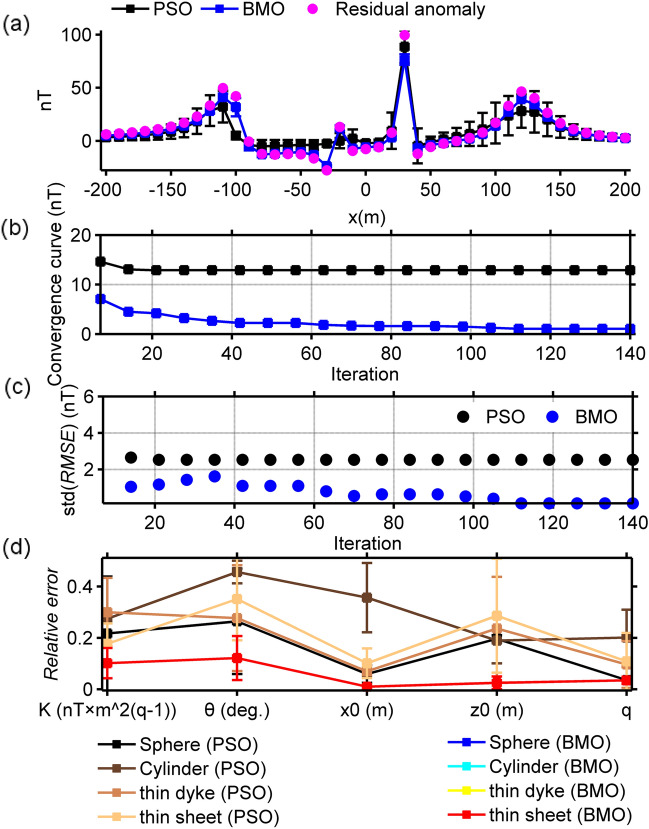
Inversion results of case 1. (**a**) fittings between the simulated residual magnetic anomaly and the responses obtained via BMO and sPSO, (**b**) the variation behavior of the mean *RMSE* values against iterations after 30 runs, (**c**) the variation behavior of the std of *RMSE* values against iterations after 30 runs, (**d**) calculated relative errors of model parameter solutions using BMO and sPSO for four causative sources. Error bars are given in (**a**) and (**d**).

The synthetic magnetic anomaly generated and the causative sources are shown in Fig. 5. We performed 30 independent runs using 80 search agents and 140 iterations for both BMO and sPSO. Table 3 lists the model parameters search spaces and the solutions obtained. Note that the run-time of sPSO and BMO were 74 s and 67 s, respectively. The magnetic anomaly responses calculated through the BMO and sPSO algorithms were compared with the residual magnetic anomaly (Fig. 6a). Figure 6b,c show the behaviors of the mean value and the std of the calculated RMSE values against the iteration number. Figure 6d shows the calculated relative errors of the model parameters recovered via BMO and sPSO approaches. As seen from Fig. 6 and Table 3, the application of BMO algorithm produced stable, accurate, and robust performance. Model parameters with larger error values were obtained with the sPSO application. On the other hand, sPSO showed a faster convergence rate when using large population of search agents. sPSO and BMO converged to global minimum within 20 and 60 iterations, respectively. Although sPSO showed a faster rate of convergence, the resulting *RMSE* values showed that BMO better achieved an adequate trade-off to balance the exploration and exploitation stages.

**Table 3:** Inversion results of the pure residual and contaminated magnetic anomaly caused by multiple-sources using BMO and PSO without and with the implementation of the SMA technique. The order of source structures is as in Table 2.

Without the implementation of the SMA technique						
Case 1						
Parameter set	True parameters	Search space	PSO results	Relative error	BMO results	Relative error
*K* (nT × m^2*q*−2^)	60/2000/50/800	30 ~ 90/1000 ~ 3000/25 ~ 75/400 ~ 1200	67.157 ± 18.836/2194.3 ± 630.56/50.053 ± 19.537/766.41 ± 180.01	0.21731 ± 0.2215/0.2747 ± 0.0177/0.2998 ± 0.1334/0.1770 ± 0.07898	**63.058** ± 7.3722/**2101.9** ± 245.79/**52.549** ± 6.1425/**840.77** ± 98.298	0.1017 ± 0.0589/0.1017 ± 0.0589/0.1017 ± 0.0589/0.1017 ± 0.0589
*θ* (degree)	60/30/10/50	30 ~ 90/15 ~ 45/5 ~ 15/25 ~ 75	64.14 ± 22.427/25.437 ± 15.857/8.8022 ± 3.6846/38.486 ± 18.097	0.2643 ± 0.2050/0.4561 ± 0.0437/0.2769 ± 0.2056/0.3509 ± 0.16049	**67.292** ± 5.124/**33.647** ± 2.5618/**11.216** ± 0.85386/**56.078** ± 4.27	0.1215 ± 0.0854/0.1215 ± 0.0853/0.1215 ± 0.0853/0.1215 ± 0.0853
*x*_0_ (m)	30/− 25/120/− 100	15 ~ 45/− 37.5 ~ − 12.5/60 ~ 180/− 150 ~ − 50	30.69 ± 2.2587/− 21.693 ± 10.679/117.39 ± 9.9492/− 110.11 ± 5.7627	0.0594 ± 0.0340/0.3564 ± 0.1345/0.0699 ± 0.0160/0.1011 ± 0.0576	**30.008** ± 0.4415/− **24.993** ± 0.3680/**120.03** ± 1.7642/− **99.975** ± 1.4717	0.0104 ± 0.0073/0.0104 ± 0.0073/0.0104 ± 0.0073/0.0104 ± 0.0073
*z*_0_ (m)	8/5/20/12	4 ~ 12/2.5 ~ 7.5/10 ~ 30/6 ~ 18	6.4271 ± 0.1128/5.9484 ± 0.4432/21.301 ± 6.8666/10.973 ± 4.8138	0.1966 ± 0.0141/0.1896 ± 0.0886/0.2363 ± 0.2011/0.2861 ± 0.2215	**8.0844** ± 0.2987/**5.053** ± 0.1864/**20.211** ± 0.7461/**12.127** ± 0.4479	0.0251 ± 0.0246/0.0251 ± 0.0247/0.0251 ± 0.0246/0.0251 ± 0.0246
*q*	2.5/2/1/1	1.25 ~ 3.75/1 ~ 3/0.5 ~ 1.5/0.5 ~ 1.5	2.4683 ± 0.1031/2.4027 ± 0.2159/1.0848 ± 0.1071/1.1085 ± 0.1109	0.035874 ± 0.0035773/0.20137 ± 0.10795/0.096601 ± 0.090866/0.1085 ± 0.11097	**2.5871** ± 0.0354/**2.0697** ± 0.0284/**1.0348** ± 0.01420/**1.0348** ± 0.0142	0.0348 ± 0.0141/0.0348 ± 0.0142/0.0348 ± 0.0142/0.0348 ± 0.0142
*RMSE* between the uncontaminated magnetic anomaly and the obtained residual responses			12.8836 ± 2.5283		**5.3159** ± 0.2323	
**Case 2**						
*K* (nT × m^2*q*−2^)			54.578 ± 34.758/1194.9 ± 275.63/30.162 ± 7.3005/444.88 ± 63.473	0.4096 ± 0.1278/0.4025 ± 0.1378/0.3967 ± 0.1460/0.4439 ± 0.07934	66.914 ± 4.0545/1693.8 ± 143.63/55.618 ± 11.24/675.77 ± 56.635	0.1152 ± 0.0675/0.1530 ± 0.0718/0.1589 ± 0.1589/0.1552 ± 0.0707
*θ* (degree)			30 ± 0/30 ± 21.213/14.145 ± 1.2094/41.359 ± 23.135	0.5 ± 0/0.5 ± 0/0.4144 ± 0.1209/0.3271 ± 0.2444	67.36 ± 13.165/24.475 ± 9.178/11.321 ± 0.58428/48.068 ± 7.3699	0.1551 ± 0.1734/0.2163 ± 0.2604/0.1320 ± 0.0584/0.1042 ± 0.0546
*x*_0_ (m)			30.529 ± 0.9287/− 28.526 ± 0.5461/155.81 ± 12.113/− 75.298 ± 35.776	0.02189 ± 0.0249/0.1410 ± 0.0218/0.298 ± 0.1009/0.2529 ± 0.3493	31.016 ± 2.3547/− 21.053 ± 6.1831/142.89 ± 14.63/− 93.811 ± 3.8708	0.0555 ± 0.0479/0.1748 ± 0.2232/0.1907 ± 0.1219/0.0618 ± 0.0387
*z*_0_ (m)			8.5367 ± 4.8979/7.5 ± 0/16.661 ± 9.4195/6 ± 0	0.4329 ± 0.0948/0.5 ± 0/0.3330 ± 0.2361/0.5 ± 0	9.1818 ± 1.5044/5.103 ± 0.5569/18.28 ± 0.88362/10.437 ± 5.0416	0.1477 ± 0.1880/0.0787 ± 0.0291/0.0860 ± 0.0441/0.2970 ± 0.1841
*q*			2.9844 ± 0.7070/2.8102 ± 0.2684/0.7169 ± 0.1668/1.2422 ± 0.1495	0.2 ± 0.2740/0.4050 ± 0.1342/0.2831 ± 0.1668/0.2421 ± 0.1495	2.7649 ± 0.1359/2.2638 ± 0.1980/0.9693 ± 0.05981/1.1929 ± 0.087333	0.1059 ± 0.05436/0.1318 ± 0.0990/0.0422 ± 0.0434/0.1929 ± 0.0873
*RMSE* between the contaminated magnetic anomaly and the obtained responses			40.9405 ± 0.0534		45.4893 ± 3.2635	
*RMSE* between the uncontaminated magnetic anomaly and the obtained residual responses			37.4692 ± 14.9365		21.7046 ± 6.7316	
**With the implementation of the SMA technique (the calculated mean results using different *s* values are given)**						
**Case 2**						
*K* (nT × m^2*q*−2^)	60/2000/50/800	30 ~ 90/1000 ~ 3000/25 ~ 75/400 ~ 1200	58.534 ± 11.944/1816.7 ± 328.01/52.685 ± 8.6997/797.78 ± 105.75	0.2365 ± 0.0746/0.2241 ± 0.0808/0.2053 ± 0.1000/0.1786 ± 0.0878	**60.623** ± 1.7993/**2037.3** ± 74.326/**48.386** ± 1.9889/**777.27** ± 43.932	0.0607 ± 0.0212/0.0638 ± 0.0373/0.0608 ± 0.0258/0.0684 ± 0.0276
*θ* (degree)	60/30/10/50	30 ~ 90/15 ~ 45/5 ~ 15/25 ~ 75	63.913 ± 7.9871/31.838 ± 5.4413/10.933 ± 2.0296/49.247 ± 9.219	0.2168 ± 0.0727/0.1842 ± 0.03944/0.1938 ± 0.0939/0.1845 ± 0.0957	**58.764** ± 4.401/**28.553** ± 2.3802/**9.7616** ± 0.79822/**48.119** ± 3.4934	0.0532 ± 0.0575/0.0772 ± 0.0568/0.0681 ± 0.0513/0.0775 ± 0.0458
*x*_0_ (m)	30/− 25/120/− 100	15 ~ 45/− 37.5 ~ − 12.5/60 ~ 180/− 150 ~ − 50	30.041 ± 0.60487/− 24.432 ± 1.2435/115.59 ± 17.989/− 98.297 ± 8.158	0.0199 ± 0.0119/0.0483 ± 0.0645/0.1867 ± 0.0797/0.0672 ± 0.0707	**29.965** ± 0.2545/− **24.801** ± 0.5644/**120.37** ± 3.3909/− **99.84** ± 2.0854	0.0193 ± 0.0080/0.0205 ± 0.0118/0.0273 ± 0.0166/0.02417 ± 0.0109
*z*_0_ (m)	8/5/20/12	4 ~ 12/2.5 ~ 7.5/10 ~ 30/6 ~ 18	7.1768 ± 1.0318/4.7307 ± 0.7216/17.265 ± 2.3129/11.51 ± 1.6127	0.2029 ± 0.0524/0.1721 ± 0.097/0.2195 ± 0.0881/0.2103 ± 0.0602	**7.9239** ± 0.4027/5**.0283** ± 0.3071/**19.856** ± 0.8801/**12.324** ± 0.578	0.0590 ± 0.0234/0.0791 ± 0.0329/0.0561 ± 0.0244/0.056511 ± 0.02568
*q*	2.5/2/1/1	1.25 ~ 3.75/1 ~ 3/0.5 ~ 1.5/0.5 ~ 1.5	2.4956 ± 0.0287/2.1173 ± 0.1719/1.0472 ± 0.0861/1.0499 ± 0.10461	0.0166 ± 0.0076/0.0849 ± 0.0607/0.1906 ± 0.0991/0.1164 ± 0.0629	**2.5159** ± 0.0232/**2.0363** ± 0.0286/**1.0108** ± 0.0194/**1.011** ± 0.0212	0.0089 ± 0.0076/0.0210 ± 0.0135/0.0283 ± 0.0227/0.0286 ± 0.0273
*RMSE* between the calculated SMA magnetic anomalies and the inverted ones			18.3725 ± 0.2184		15.8042 ± 0.5604	
*RMSE* between the uncontaminated magnetic anomaly and the obtained residual responses			23.9215 ± 10.1662		**5.5187** ± 0.8829	
**Case 3**						
*K* (nT × m^2*q*^^−^^2^)			64.628 ± 13.693/2251 ± 214.83/50.535 ± 5.3922/811.02 ± 205.46	0.2133 ± 0.0741 /0.2104 ± 0.0787/0.2299 ± 0.0646/0.2368 ± 0.0511	**60.135** ± 3.6594/**2005.6** ± 168.49/**50.706** ± 2.5656/**788.2** ± 63.652	0.0823 ± 0.0498/0.0760 ± 0.0312/0.0741 ± 0.0199/0.0748 ± 0.0271
*θ* (degree)			66.58 ± 8.3505/31.9 ± 6.9616/10.052 ± 1.3155/54.51 ± 8.1819	0.2119 ± 0.0837/0.2022 ± 0.0979/0.1883 ± 0.0935/0.2179 ± 0.0574	**59.292** ± 3.9508/**29.679** ± 1.3942/**9.884** ± 0.26552/**49.094** ± 3.2371	0.0618 ± 0.0380/0.0493 ± 0.0324/0.0408 ± 0.0203/0.06890 ± 0.0390
*x*_0_ (m)			29.58 ± 0.68447/− 25.127 ± 1.1433/127.74 ± 21.46/− 91.905 ± 10.83	0.0277 ± 0.0219/0.0491 ± 0.0385/0.1754 ± 0.119/0.0810 ± 0.1082	**29.978** ± 0.5206/− **24.901** ± 0.7025/**120.3** ± 3.157/− **99.408** ± 0.4812	0.0217 ± 0.0082/0.0311 ± 0.0167/0.0302 ± 0.0183/0.01188 ± 0.01176
*z*_0_ (m)			8.683 ± 0.6031/5.3544 ± 0.7323/19.928 ± 2.6287/11.909 ± 1.2843	0.1785 ± 0.0674/0.1855 ± 0.0517/0.1735 ± 0.0820/0.1779 ± 0.05247	**8.2001** ± 0.3039/**5.0115** ± 0.2916/**19.23** ± 1.3641/**12.113** ± 0.32479	0.0636 ± 0.0105/0.0704 ± 0.0345/0.0660 ± 0.0548/0.0566 ± 0.0383
*q*			2.5001 ± 0.0561/2.0916 ± 0.1450/1.0016 ± 0.1319/1.0419 ± 0.1449	0.0186 ± 0.0133/0.074481 ± 0.0449/0.15597 ± 0.0725/0.1537 ± 0.0612	**2.5211** ± 0.0486/**2.0138** ± 0.0402/**0.9876** ± 0.0299/**0.9944** ± 0.0207	0.0160 ± 0.0124/0.0188 ± 0.0097/0.0339 ± 0.0221/0.02533 ± 0.0171
*RMSE* between the calculated SMA magnetic anomalies and the inverted ones			23.1848 ± 0.8570		21.2171 ± 0.4926	
*RMSE* between the uncontaminated magnetic anomaly and the obtained residual responses			28.1128 ± 6.7413		**7.0089** ± 2.5616	

Significant values are in bold.

#### Case 2

**Figure 7:**
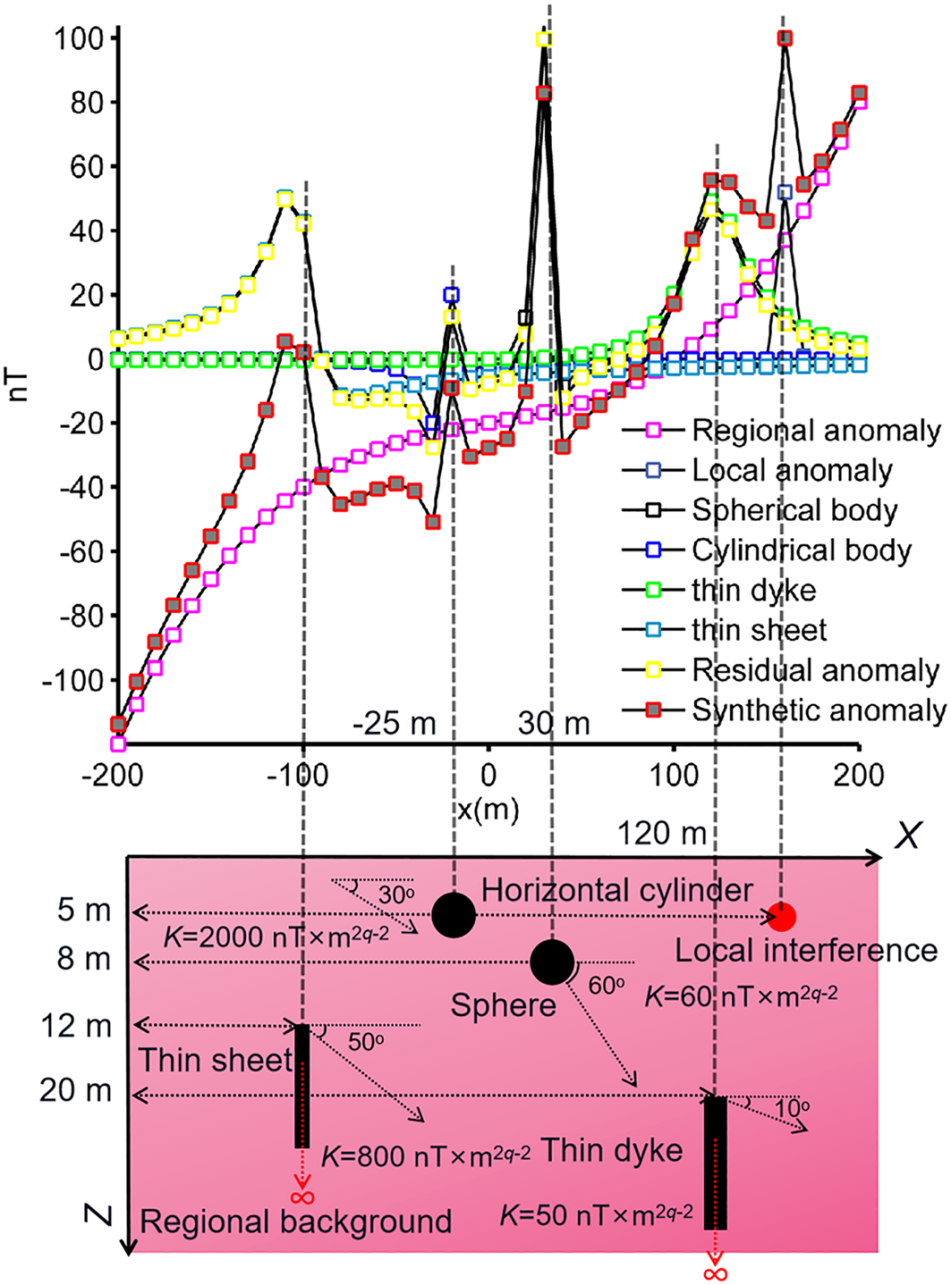
Synthetic residual magnetic anomaly of the multiple-source model (case 2) superposed on a regional background and local interference, and the causative subsurface model.

**Figure 8:**
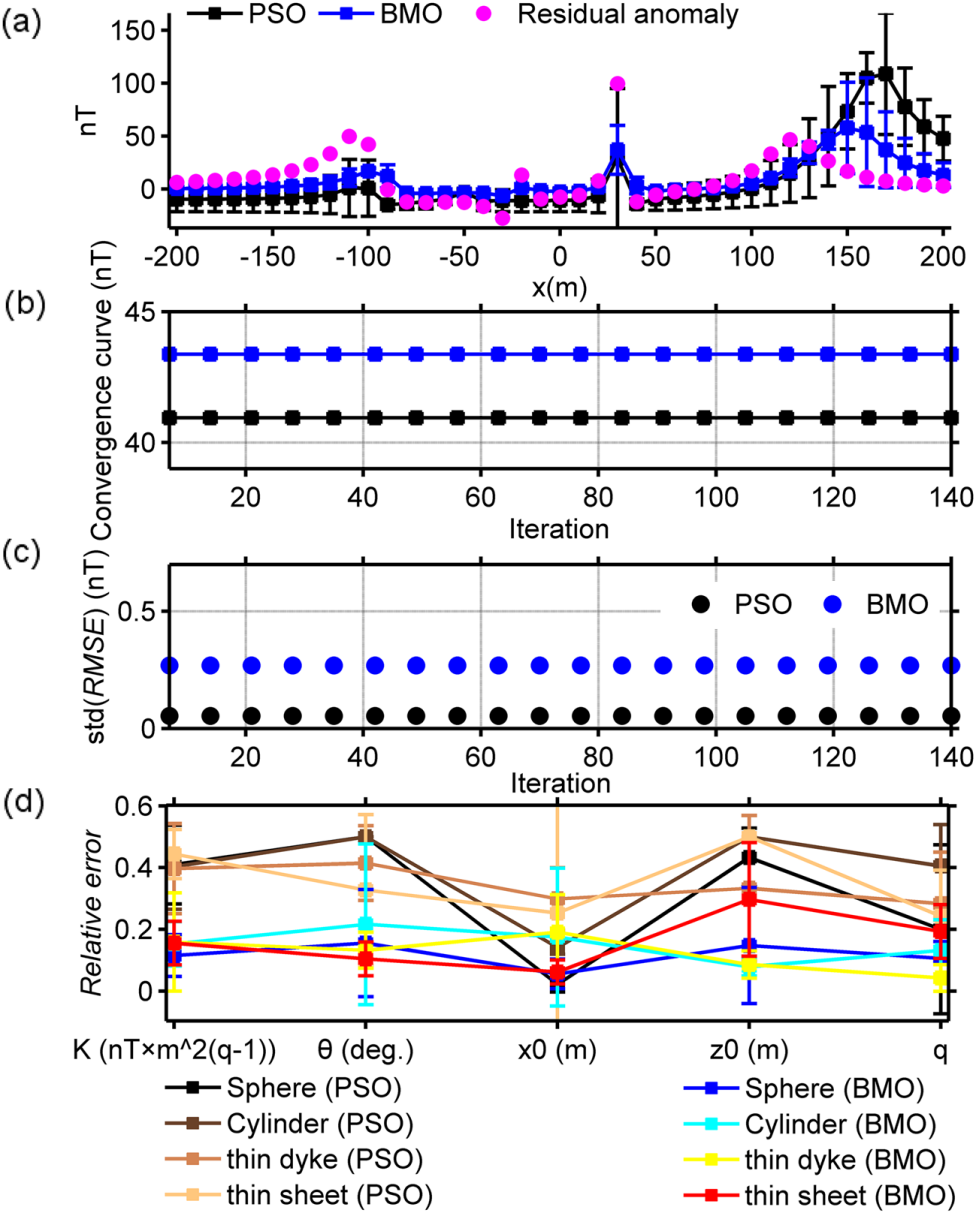
Inversion results of case 2 without the implementation of the SMA technique. (**a**) fittings between the pure residual magnetic anomaly and the responses obtained via BMO and sPSO, (**b**) the variation behavior of the mean *RMSE* values against iterations after 30 runs, (**c**) the variation behavior of the std of *RMSE* values against iterations after 30 runs, (**d**) calculated relative errors of model parameter solutions using BMO and sPSO for four causative sources. Error bars are given in (**a**) and (**d**).

**Figure 9:**
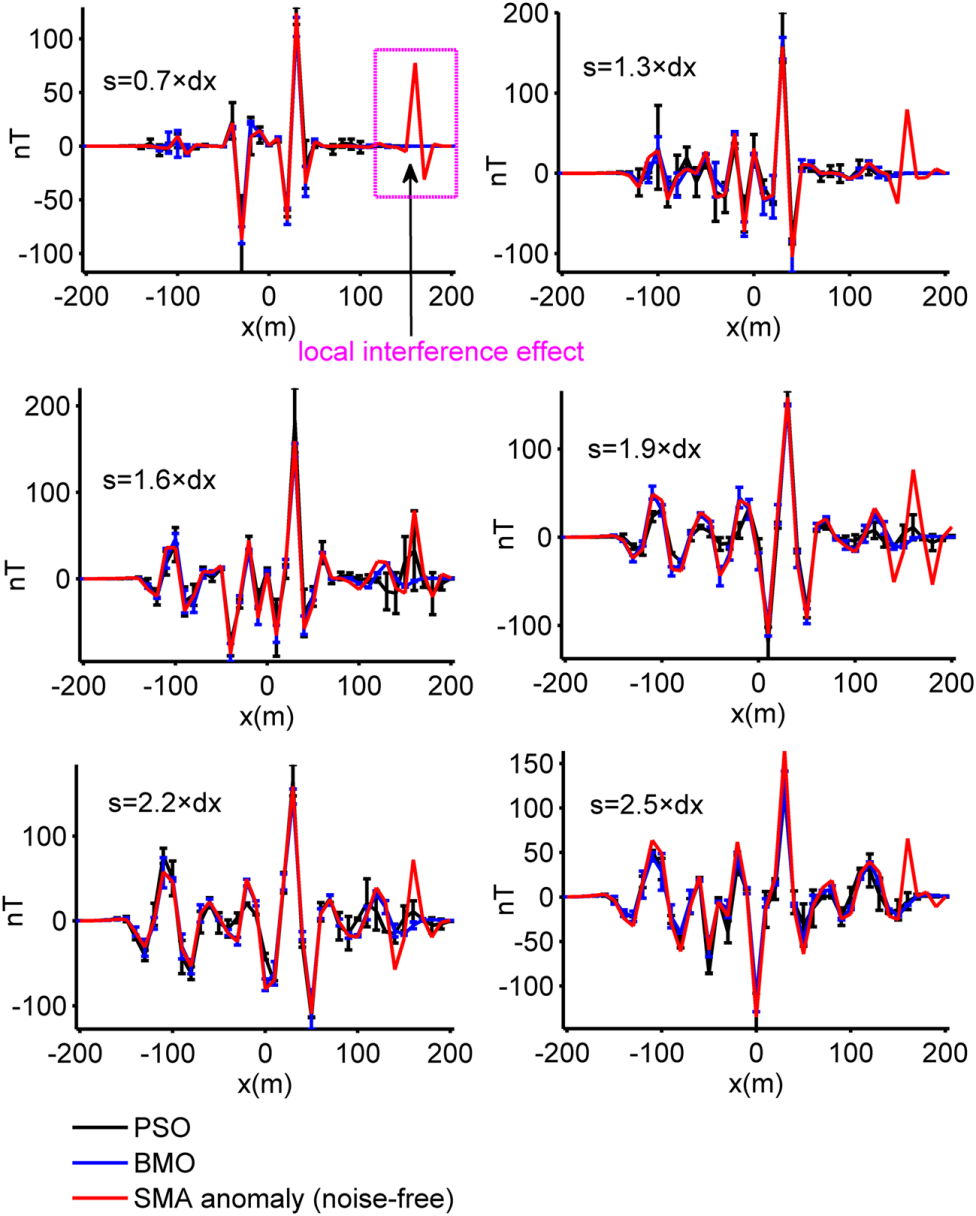
The calculated SMA magnetic anomalies of case 2 and estimated SMA magnetic responses using BMO and sPSO for different *s* values (0.7, 1.3, 1.6, 1.9, 2.2, and 2.5 × dx, dx = 10 m).

**Figure 10:**
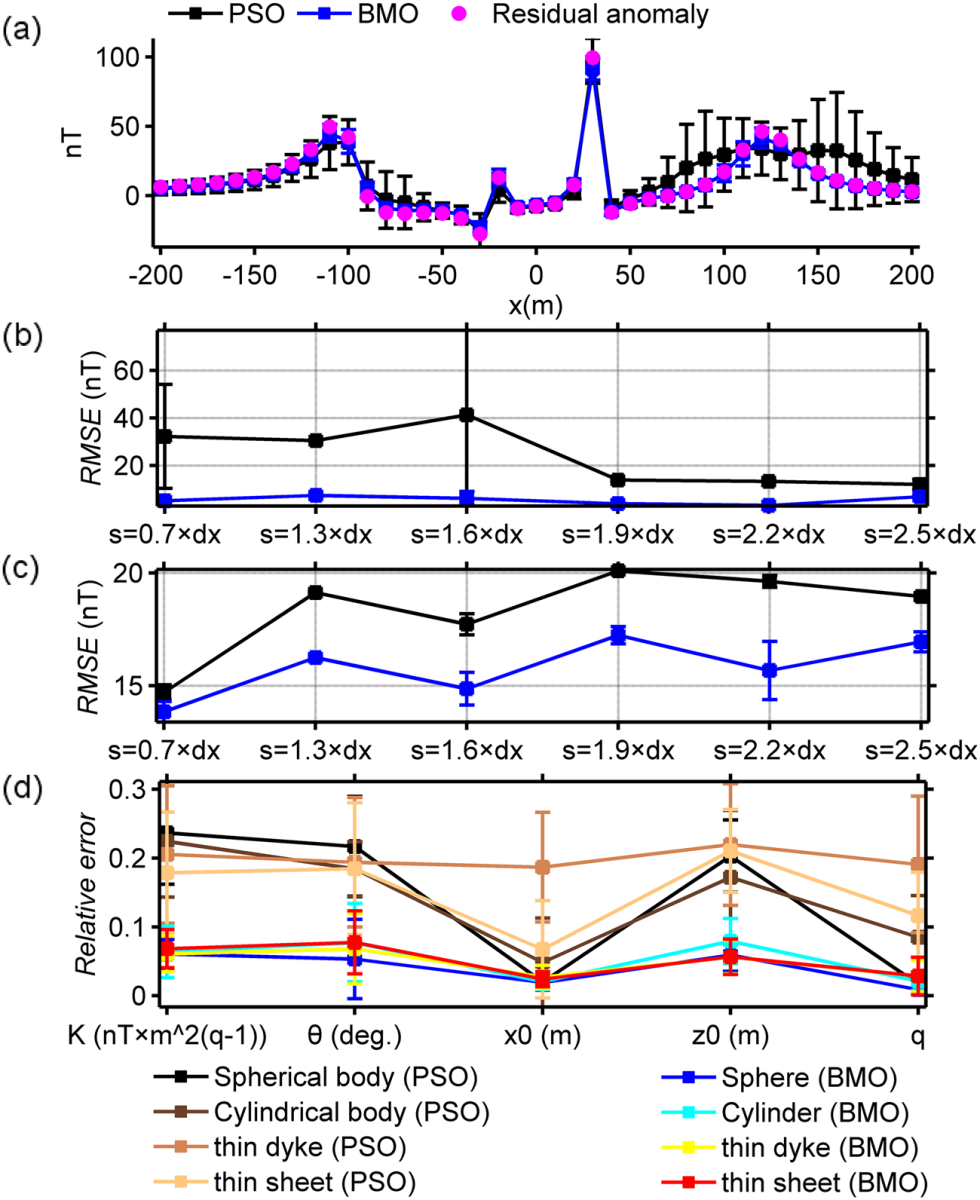
Inversion results of case 2 with the implementation of the SMA technique. (**a**) fittings between the pure residual magnetic anomaly and the responses obtained via BMO and sPSO, (**b**) variation behavior of the calculated *RMSE* values between the uncontaminated magnetic anomaly of case 1 and the residual responses obtained via BMO and sPSO for different *s* values, (**c**) variation behaviors of the computed *RMSE* values between the calculated SMA magnetic anomalies of case 2 and the estimated SMA magnetic responses of BMO and sPSO for different *s* values, (**d**) relative errors of the calculated average estimation of the obtained model parameters using BMO and sPSO for four causative sources from the SMA magnetic anomalies for several *s* values. Error bars are given in (**a**)–(**d**).

In this experiment we added regional and local effects to the synthetically generated magnetic anomaly using the following expressions:

16 $$T_{gen}^{2} (x_{i} ) = T_{p = 4}^{1} (x_{i} ){ + }T_{re} (x_{i} ){ + }T_{local} (x_{i} ),$$

17 $$T_{re} (x_{i} ) = 10^{ - 6} x_{i}^{3} + 10^{ - 7} x_{i}^{2} + 10^{ - 2} x_{i} - 20,$$

18 $$T_{local} (x_{i} ) = 30 \times 5^{3} \left\{ {\frac{{\left[ {2 \times 5^{2} - \left( {x_{i} - 160} \right)^{2} } \right] \times \sin 120^\circ + 3 \times 5 \times \left( {x_{i} - 160} \right) \times \cos 120^\circ }}{{\left[ {\left( {x_{i} - 160} \right)^{2} + 5^{2} } \right]^{5/2} }}} \right\},$$

where $T_{gen}^{2} (x_{i} )$ denotes the composite anomaly, *T*_*re*_(*x*_*i*_) is the regional background effect and *T*_*local*_(*x*_*i*_) represents the local perturbation originated from a near-surface spherical interference. Figure 7 illustrates the subsurface magnetized sources and the resulting magnetic responses. BMO and sPSO were applied using 30 independent runs with 80 search agents and 140 iterations. The control parameters of both optimizers were kept unchanged, and applications were performed without using the SMA technique. Search space bounds consistent with the first case were used. After each iteration, the mean, std of *RMSE* values, and the relative errors of estimated parameters were recorded to observe the performances (Fig. 8). Table 3 stores the quantitative results of estimations. The run-time of sPSO and BMO algorithms were 69 s and 66 s, respectively. It is clear that the mean *RMSE* value increased significantly with the final iteration. Moreover, the error values between the calculated and the true model parameters of four causative sources increased too. It is clear that both algorithms underperformed due to the presence of external effects incorporated. Subsequently, we experienced the effectiveness of the SMA technique in reducing the regional effects. Figure 9 illustrates the calculated SMA magnetic anomalies (red lines) for some *s* values (0.7, 1.3, 1.6, 1.9, 2.2, and 2.5 × dx, dx equals the data spacing interval). Blue and black lines represent the calculated responses obtained through BMO and sPSO, respectively and are generally in well agreement with the red lines except for the anomalies generated from the interfering shallow spheric source. In this scenario, the running times of the sPSO and BMO algorithms are equal to the product of the times given previously and the s-values used, namely 69 s and 66 s. Hence, we will solely present the computational time considering the circumstance of delineating the residual anomaly. Outputs of this synthetic experiment and the detailed inversion results are shown in Fig. 10 and Table 3, respectively. It is clear that the SMA technique proved useful in eliminating the regional effect from the composite magnetic anomaly and therefore both algorithms yielded relatively agreeable results. However, examining the results in detail, it is seen that the mathematical nature of simulating the special mating behaviors of barnacles yielded lower fitness errors, more accurate model parameter values, and higher inversion stability than sPSO.

#### Case 3

**Figure 11:**
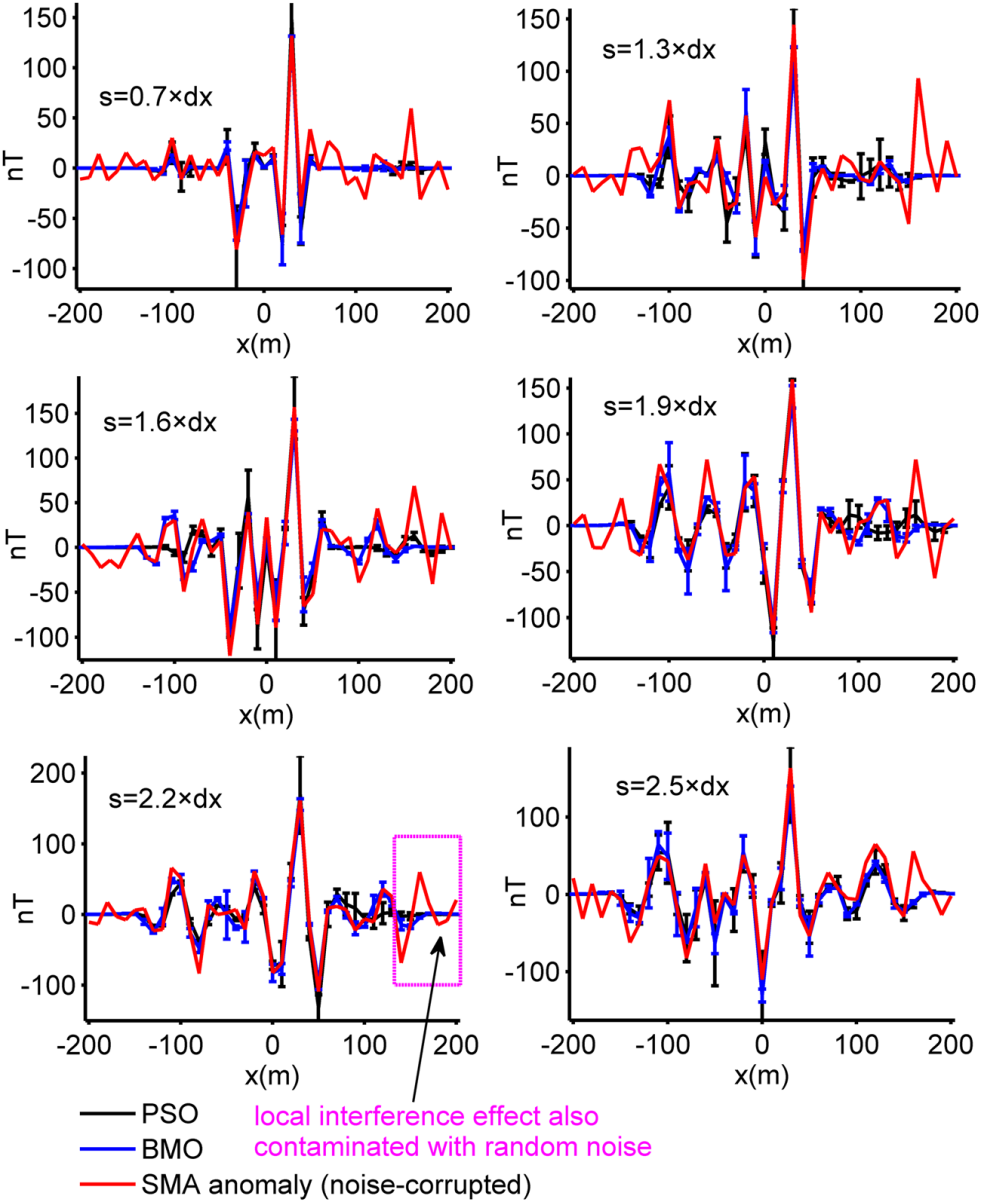
The SMA results of 40% random noise-corrupted magnetic anomalies of case 2 and estimated SMA magnetic responses via BMO and sPSO for different *s* values (0.7, 1.3, 1.6, 1.9, 2.2, and 2.5 × dx, dx = 10 m).

**Figure 12:**
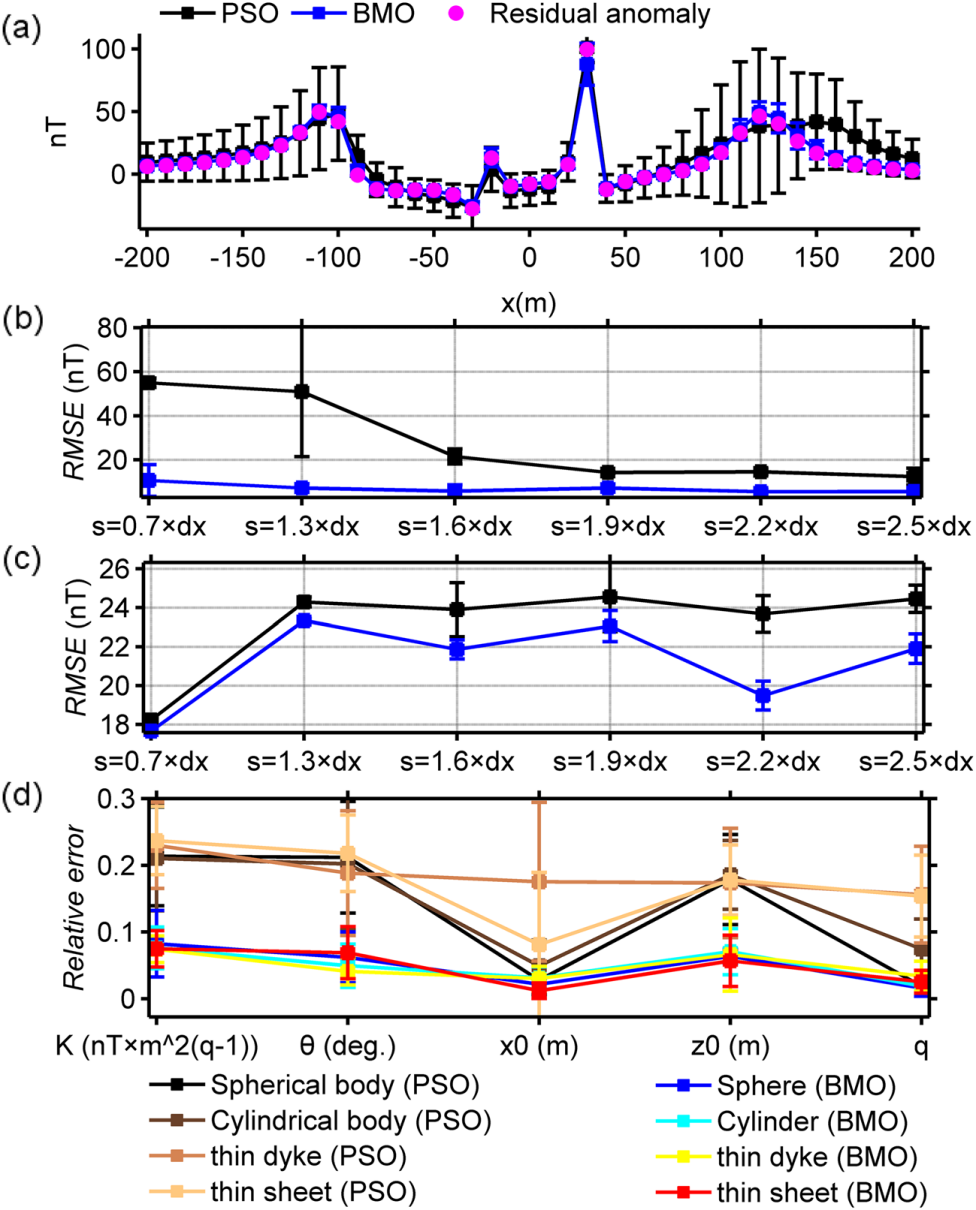
Inversion results of case 3 with the implementation of the SMA technique. (**a**) fittings between the residual magnetic anomaly and the responses obtained via BMO and sPSO, (**b**) variation behaviors of the calculated *RMSE* values between the uncontaminated magnetic anomaly of case 1 and the residual responses obtained via BMO and sPSO for different *s* values, (**c**) variation behaviors of the computed *RMSE* values between the calculated SMA magnetic anomalies of case 3 and the estimated SMA magnetic responses of BMO and sPSO for different *s* values, (**d**) relative errors of the calculated average estimation of the obtained model parameters using BMO and sPSO for four causative sources from the SMA magnetic anomalies for different *s* values. Error bars are also given in (**a**)–(**d**).

In order to make the work of both algorithms more difficult, a noise content of 40% was added to the magnetic anomaly of case 2 by using the definition below:

19 $$\widetilde{T}_{gen}^{2} (x_{i} ) = T_{gen}^{2} (x_{i} ){ + 0}{\text{.4}} \times {\text{mean}}\left( {\left| {T_{gen}^{2} (x)} \right|} \right) \times \left[ {{\text{rand}}_{1} ({\text{M}}) - {\text{rand}}_{2} ({\text{M}})} \right],$$

where mean() is the average value of the input, rand_1_() and rand_2_() return an array containing pseudorandom values between [0, 1] of a given size. Using the same computation procedures, we inverted the synthetic magnetic anomaly through both algorithms. The SMA noisy anomaly and estimated SMA magnetic responses obtained by means of BMO and sPSO (blue and black solid lines) for different *s* values are demonstrated in Fig. 11. Figure 12 and Table 3 show the outputs of the optimization via both algorithm and detailed inversion results, respectively. Applications showed that the added 40% random noise only produced minor perturbations to the SMA residual anomalies (red lines in Fig. 11). Both algorithms produced satisfactory solutions, but the details revealed the superiority of BMO in terms of lower error values, more accurate model parameter values, and more stable optimization.

### Post-inversion uncertainty appraisal analysis of synthetic case

**Figure 13:**
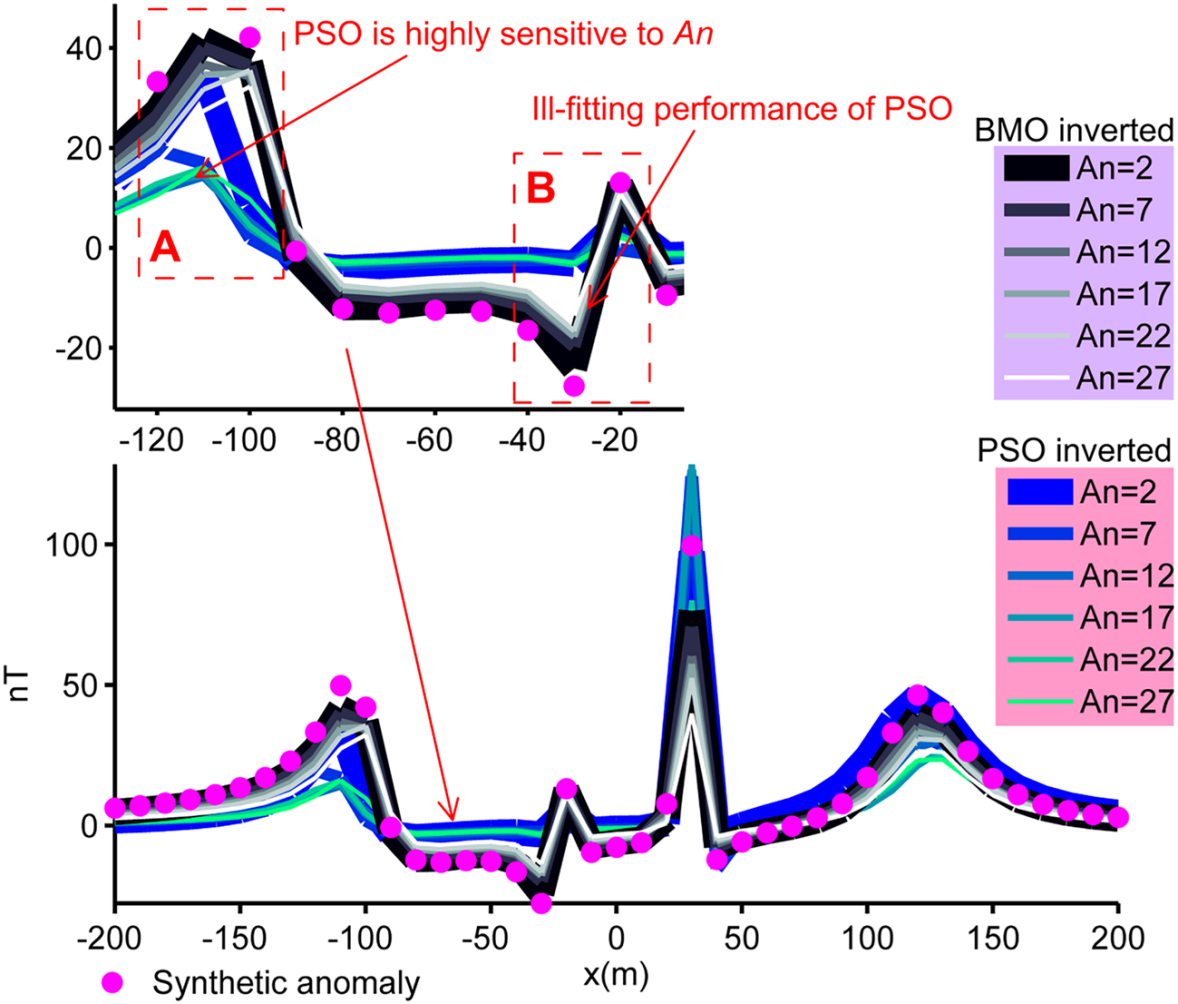
Post-inversion uncertainty appraisal analyses. Calculated mean responses of both algorithms varying with *An* (case 1).

As mentioned previously in the modal analysis section, the optimization problem presented here is mostly unstable and error-prone because of the extreme complexity of escaping massive local minima while effectively exploiting the unique global minimum. Therefore, post-inversion uncertainty assessment analyses are crucial for understanding the credibility of the model parameters recovered. To practice the uncertainty appraisal studies, we used the solutions obtained in the first case experiment. Best model parameters obtained in each independent runs were listed and then sorted in ascending order of their misfit values. We assumed *An* as a variable in order to determine the number of sorted parameters. That we used to compute the final results by calculating the mean responses. We considered *An* as a variable to determine the number of parameters sorted. We then calculated the mean of each sorted parameter values. Lastly, the mean magnetic anomaly response was obtained with the mean model parameter set. Figure 13 exhibits that BMO showed low sensitivity to the increment of *An*, however, the performance of sPSO was significantly affected with the use of larger *An* values (pointed part A). Consistently, this phenomenon is correlated with the larger std values in the inversion results of sPSO. On the other hand, the superiority of the anomaly-fitting ability of BMO regardless the value of *An* is clearly observed in pointed part B (Fig. 13). These findings clearly revealed that BMO is more capable than sPSO in obtaining more robust solutions for this inverse problem presented.

## Real data applications

The performance of BMO in the inversion of real magnetic anomalies was tested using three field cases from India and Brazil. These magnetic anomalies were considered as residual responses and were studied by some researchers using several data processing techniques. Based on this information, we initially treated these data sets as residual magnetic anomalies and applied the BMO algorithm using *pl* = 0.65 × *N*. We applied sPSO using the same algorithm-based control parameters to obtain outputs for the comparison tests. In the experiments, both algorithms were run independently 30 times using 80 search agents and 140 iterations. We then applied BMO procedure to SMA anomalies to understand if these anomalies are contaminated by regional background effect and/or local interference. Finally, the model parameter solutions obtained were interpreted in an integrated manner with the known geological data and/or previous geophysical estimates given in Table 4.

**Table 4:** Reported results of the field cases.

Chromite Ore, Tangarparha, Odisha, India					
Parameter set	*K* (nT × m^2*q*−2^)	*x*_0_ (m)	*z*_0_ (m)	*θ* (degree)	*q*
Biswas (2018)	− 118.93 ± 0.69/370.01 ± 05/− 143.22 ± 1.04	45.77 ± 0.57/825.20 ± 0.20/1062.16 ± 1.22	150.36 ± 0.31/344.30 ± 0.70/261.43 ± 2.17	163.75 ± 0.34/0.00 ± 0.5/4.64 ± 0.39	2.5/2.5/2.5
**Uranium Ore, Beldih Mine, Purulia, West Bengal, India**					
Biswas (2018)	779.86 ± 1.17/3183.15 ± 8.71/1760.56 ± 10.80	110.74 ± 0.03/166.14 ± 0.21/255.71 ± 1.38	8.39 ± 0.02/34.26 ± 0.10/25.72 ± 0.67	57.95 ± 0.15/54.89 ± 0.37/− 2.45 ± 4.83	1/1/1
**Parnaiba anomaly, Brazil**					
Biswas (2018)	− 1541.27 ± 16.05	0.57 ± 0.02	3.47 ± 0.03	59.24 ± 0.30	2
Tlas and Asfahani (2015)	− 645.6	0	3.4	41.3	2
Abdelrahman and Essa (2015)	–	–	2.35	–	1.02
Asfahani and Tlas (2007)	− 59.8	–	2.3	47.1	–
Asfahani and Tlas (2004)	− 59.81	–	2.26	47.11	–
Abdelrahman and Sharafeldin (1996)	− 58.6	–	3.5	33.3	–
Silva (1989)	–	–	3.5	–	–

### Chromite Ore Anomaly, India

**Figure 14:**
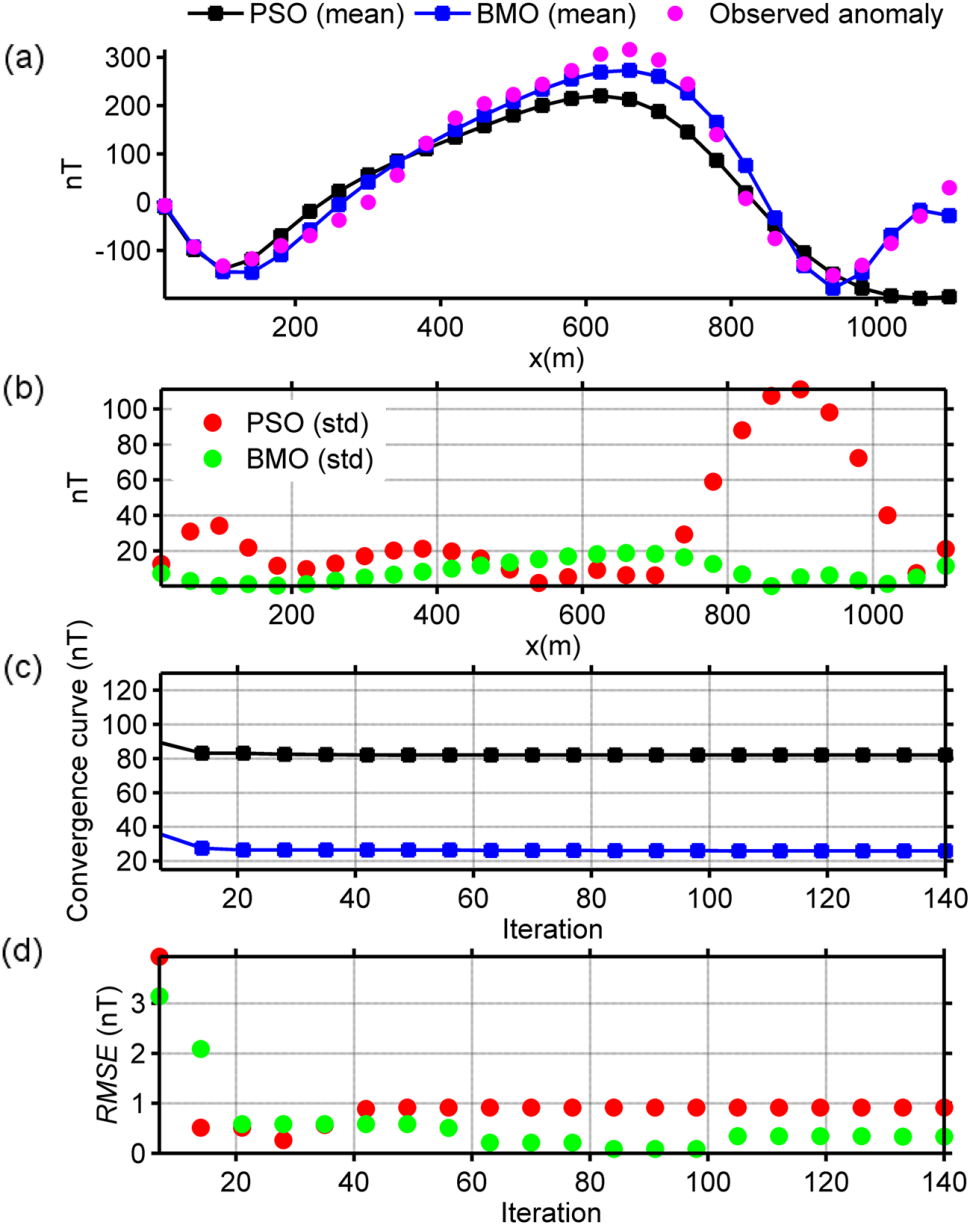
Inversion results of the chromite ore anomaly, India. (**a**) fittings between the observed magnetic anomaly and the mean responses obtained via BMO and sPSO, (**b**) calculated std values of obtained responses at each station, (**c**) variation behavior of the mean *RMSE* values against iterations of BMO and sPSO after 30 independent runs, (**d**) variation behavior of the std of *RMSE* values against iterations of BMO and sPSO after 30 runs.

The studied chromite ores are located in Tangarparha, Odisha, India. According to *prior* information, the ore bodies in this area are known to have a pod-type structure that is more reminiscent of a sphere-shaped source^12^. The magnetic anomaly response^12,76^ of these magnetized sources is presented in Fig. 14a with pink circles. The data sampling interval is 40 m for this anomaly. This anomaly was studied previously by using Very Fast Simulated Annealing (VFSA) technique with various source assumptions such as single spherical-shaped body, cylinder, dyke, and sheet^12^. This magnetic anomaly was interpreted to originate from three spherical source bodies^12^. Model parameter solutions revealed in that study are given in Table 4. First, we considered this finding and applied the BMO and sPSO algorithms to estimate the model parameters. Search space bounds for model parameters are given in Table 5. Figure 14a displays the comparison between the observed data and the reconstructed mean responses. Figure 14b shows the calculated std values of obtained responses at each station. The mean value and std of *RMSE* against the iterations for 30 independent runs are shown in Fig. 14c,d. Table 5 lists the obtained model solutions (*K*, *θ*, *x*_0_, *z*_0_, *q*) and *RMSE*s. The run-time of sPSO and BMO were 65 s and 59 s, respectively. The inversion results indicated that the assumed three spherical bodies approximate the sources effectively. Despite the faster convergence rate of sPSO, BMO outperformed in terms of response-fitting and stability. In the next step, we applied BMO to the SMA anomaly (*s* = 0.5, 0.75, and 1 × dx, dx = 40 m). Figure 17a displays the SMA results (red lines) for various *s* values and obtained responses (blue lines). Notably, model parameters obtained using the SMA technique (Table 5) are in well agreement with the results accomplished from considering the acquired data as a pure residual response. This finding indicates that the observed magnetic response does not contain undesired structural information such as regional background and near-surface source effects, but may contain random noise. The solutions obtained here are relatively in well agreement with the ones achieved via VFSA (Table 5).

**Table 5:** Inversion results of the field magnetic anomalies using BMO and PSO without and with the implementation of the SMA technique.

Chromite Ore, Tangarparha, Odisha, India			
Without the implementation of the SMA technique			
Parameter set	Search spaces	PSO results	BMO results
*K* (nT × m^2*q*−2^)	− 124.88 ~ − 41.626/230.91 ~ 692.74/− 150.15 ~ − 50.05	− 89.911 ± 0/532.39 ± 33.388/− 108.11 ± 0	− **83.456** ± 0.5482/**460.69** ± 3.0421/− **100.35** ± 0.6592
*θ* (degree)	80.154 ~ 240.46/0 ~ 0/1.2652 ~ 3.7957	156.14 ± 0.8431/0 ± 0/2.7927 ± 0.7742	**158.86** ± 1.115/**0** ± 0/**2.5077** ± 0.017604
*x*_0_ (m)	24.259 ~ 72.777/477.53 ~ 1432.6/494.74 ~ 1484.2	49.481 ± 0.9878/841.03 ± 56.92/1081.3 ± 317.91	**48.976** ± 0.1202/**964.07** ± 2.3662/**998.81** ± 2.4534
*z*_0_ (m)	112.4 ~ 337.21/254.18 ~ 762.53/103.96 ~ 311.89	181.3 ± 27.483/457.75 ± 129.73/272.66 ± 2.5461	**224.57** ± 1.906/**507.82** ± 4.3115/**207.7** ± 1.7627
*q*	1.213 ~ 3.6391/1.2523 ~ 3.757/1.1962 ~ 3.588	2.4537 ± 0.0038/2.5574 ± 0.0048/2.8354 ± 0.0289	**2.43** ± 0.0042/**2.5087** ± 0.0043/**2.3961** ± 0.0042
*RMSE* between the observed magnetic anomaly and the obtained residual responses		82.0409 ± 0.9173	**29.6877** ± 0.6917
**With the implementation of the SMA technique (the mean output of various dx)**			
*K* (nT × m^2*q*−2^)			− **83.42** ± 0.2328/**508.09** ± 1.3828/− **100.18** ± 0.2201
*θ* (degree)			**160** ± 0.4774/**0** ± 0/**1.2748** ± 0.0046261
*x*_0_ (m)			**38.92** ± 0.12763/**968.77** ± 3.514/**993.73** ± 3.3938
*z*_0_ (m)			**320.11** ± 1.6701/**540.34** ± 2.2061/**247.09** ± 1.1117
*q*			**2.4318** ± 0.0187/**2.5016** ± 0.0195/**2.3785** ± 0.0197
*RMSE* between the calculated SMA magnetic anomalies and the inverted one			4.2777 ± 0.0010
**Uranium Ore, Beldih Mine, Purulia, West Bengal, India**			
**Without the implementation of the SMA technique**			
*K* (nT × m^2*q*−2^)	500.71 ~ 1502.1/1887 ~ 5660.9/761.75 ~ 2285.2	1072.1 ± 174.09/3614.6 ± 1287.4/1934.5 ± 293.66	**1018.1** ± 1.5033/**3836.9** ± 5.7627/**1548.9** ± 2.2656
*θ* (degree)	18.261 ~ 54.783/30.131 ~ 90.393/− 1.8007 ~ − 0.600	40.125 ± 3.3586/61.178 ± 24.724/− 1.1525 ± 0	**36.585** ± 0.0702/**60.365** ± 0.1153/− **1.1984** ± 0.0023
*x*_0_ (m)	55.468 ~ 166.4/83.652 ~ 250.96/129.37 ~ 388.11	98.389 ± 26.253/165.61 ± 12.337/221.96 ± 53.706	**110.61** ± 0.2385/**166.81** ± 0.3524/**257.97** ± 0.5643
*z*_0_ (m)	6.97 ~ 20.91/16.086 ~ 48.258/5.8273 ~ 17.482	15.849 ± 5.9752/44.571 ± 2.4841/7.459 ± 0	**13.957** ± 0.2744/**32.21** ± 0.6317/**11.669** ± 0.22926
*q*	0.4635 ~ 1.3908/0.5155 ~ 1.5466/0.57816 ~ 1.7345	1.0296 ± 0.15337/1.0319 ± 0.0465/1.3087 ± 0.0335	**0.9303** ± 0.0010/**1.0346** ± 0.0012/**1.1602** ± 0.00125
*RMSE* between the observed magnetic anomaly and the obtained residual responses		262.6028 ± 63.7970	**119.7986** ± 2.7188
**With the implementation of SMA technique (the mean output of various dx)**			
*K* (nT × m^2*q*−2^)			**1135.2** ± 5.4598/**4474.1** ± 32.224/**1402.1** ± 7.4276
*θ* (degree)			**32.261** ± 0.1291/**55.003** ± 0.1336/− **0.8413** ± 0.0025
*x*_0_ (m)			**111.21** ± 0.1963/**168.96** ± 0.5606/**259.28** ± 1.4854
*z*_0_ (m)			**13.646** ± 0.0696/**33.115** ± 0.1668/**11.665** ± 0.0575
*q*			**0.9786** ± 0.0012/**1.0582** ± 0.0046/**1.1005** ± 0.0036
*RMSE* between the calculated SMA magnetic anomalies and the inverted one			41.0148 ± 0.1821
**Mesozoic dyke anomaly, Brazil**			
**Without the implementation of the SMA technique**			
*K* (nT × m^2*q*−2^)	− 2003.3 ~ − 1078.7	− 1195.3461 ± 160.9861	− **1518.7165** ± 4.3658
*θ* (degree)	41.468 ~ 77.012	41.7885 ± 0.1377	**36.0851** ± 0.1544
*x*_0_ (m)	0.399 ~ 0.741	0.6333 ± 0.0131	**0.6598** ± 0.0590
*z*_0_ (m)	2.429 ~ 4.511	4.5077 ± 0.0025	**5.7835** ± 0.0166
*q*	1.4 ~ 2.6	2.0248 ± 0.0348	**1.9700** ± 0.0030
*RMSE* between the observed magnetic anomaly and the obtained residual responses		7.8110 ± 0.0174	**4.7079** ± 0.0442
**With the implementation of the SMA technique (the mean output of various dx)**			
*K* (nT × m^2*q*−2^)			− **1531.4377** ± 84.6412
*θ* (degree)			**32.3969** ± 0.8404
*x*_0_ (m)			**0.84221** ± 0.0061
*z*_0_ (m)			**5.2524** ± 0.0488
*q*			**2.1250** ± 0.0342
*RMSE* between the calculated SMA magnetic anomalies and the inverted one			1.0191 ± 0.0001

Significant values are in bold.

### Uranium Ore Anomaly, India

**Figure 15:**
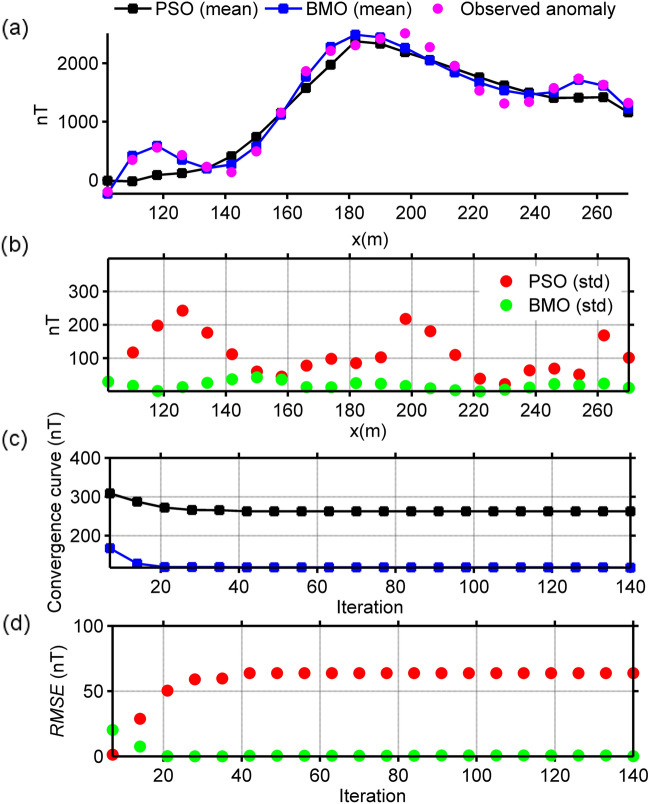
Inversion results of the uranium ore anomaly, India. (**a**) fittings between the observed magnetic anomaly and the mean responses obtained via BMO and sPSO, (**b**) calculated std values of obtained responses at each station, (**c**) variation behavior of the mean *RMSE* values against iterations of BMO and sPSO after 30 independent runs, (**d**) variation behavior of the std of *RMSE* values against iterations of BMO and sPSO after 30 runs.

The magnetic anomaly sampled at 8 m interval in the Beldih mine (Purulia, West Bengal, India) is caused from uranium ore bodies^77^. Some previous studies revealed that the uranium ore bodies in this part can be represented with vertical thick-sheet-like structures as deciphered from spontaneous-potential data interpretation^78–80^. This interpretation was validated by drilling results^81^, which disclosed that mineralization begins from the near-surface and extends to the depths of 10–20 m and which are approximately vertical and dipping northerly to southerly^12^. Additionally, gravity and resistivity responses were also used to understand subsurface nature of the source structures^77,82,83^. Lastly, this magnetic anomaly was interpreted^12^ using the VFSA technique considering dyke-like source as equal to thick-sheet-type source (Table 4). Similarly, we considered it as a residual response due to multiple dyke-like structures and implemented BMO and sPSO algorithms for reinterpretation. The model parameter ranges of assumed dykes and the results obtained are listed in Table 5. The run-time of sPSO and BMO were 54 s and 48 s, respectively. The comparison of the calculated magnetic responses with the observed one is illustrated in Fig. 15a. Figure 15b depicts the calculated std values of the obtained responses at each measurement point. Figure 15c,d demonstrates the mean value and std values of *RMSE* against the iteration number. It can be clearly seen that the anomaly of the assumed multiple dyke-like structures matches well with the observed one and BMO provides appealing results in terms of escaping local minima, fitting errors and stability of the inversion compared to those obtained via sPSO. Figure 17b illustrates the SMA anomalies (red lines) for *s* = 0.5, 0.75, and 1 × dx (dx = 8 m) and reconstructed responses via BMO (blue lines). An obvious difference between the calculated and the reconstructed responses is seen. Nevertheless, recovered model parameters given in Table 5 are correspond to the solutions obtained without the implementation of the SMA scheme. Thus, it was verified that the observed anomaly is most likely corrupted by the local interference effect and/or random noise.

### Mesozoic dyke Anomaly, Brazil

**Figure 16:**
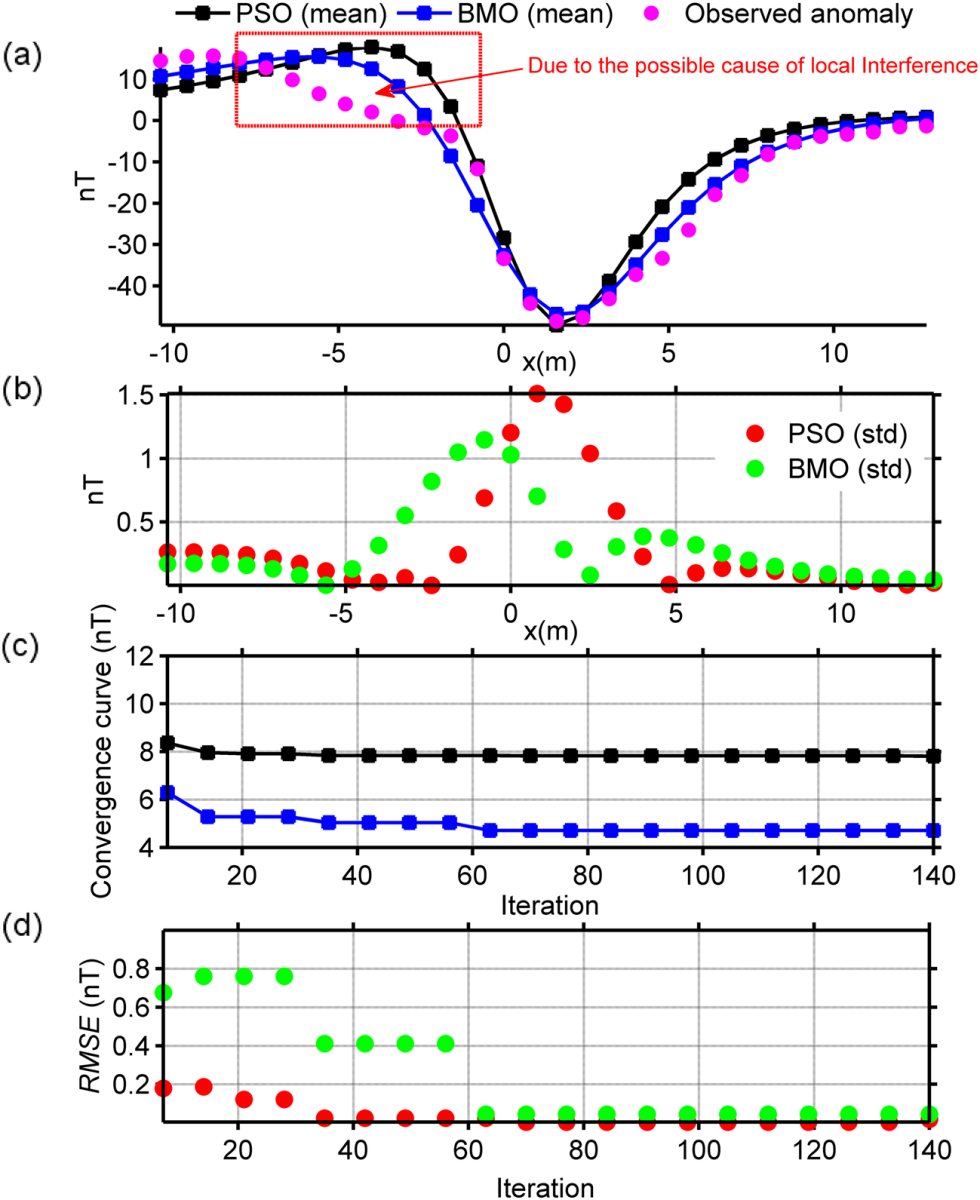
Inversion results of the Mesozoic dyke anomaly, Brazil. (**a**) fittings between the observed magnetic anomaly and the mean responses obtained via BMO and sPSO, (**b**) calculated std values of obtained responses at each station, (**c**) variation behavior of the mean *RMSE* values against iterations of BMO and sPSO after 30 independent runs, (**d**) variation behavior of the std of *RMSE* values against iterations of BMO and sPSO after 30 runs.

**Figure 17:**
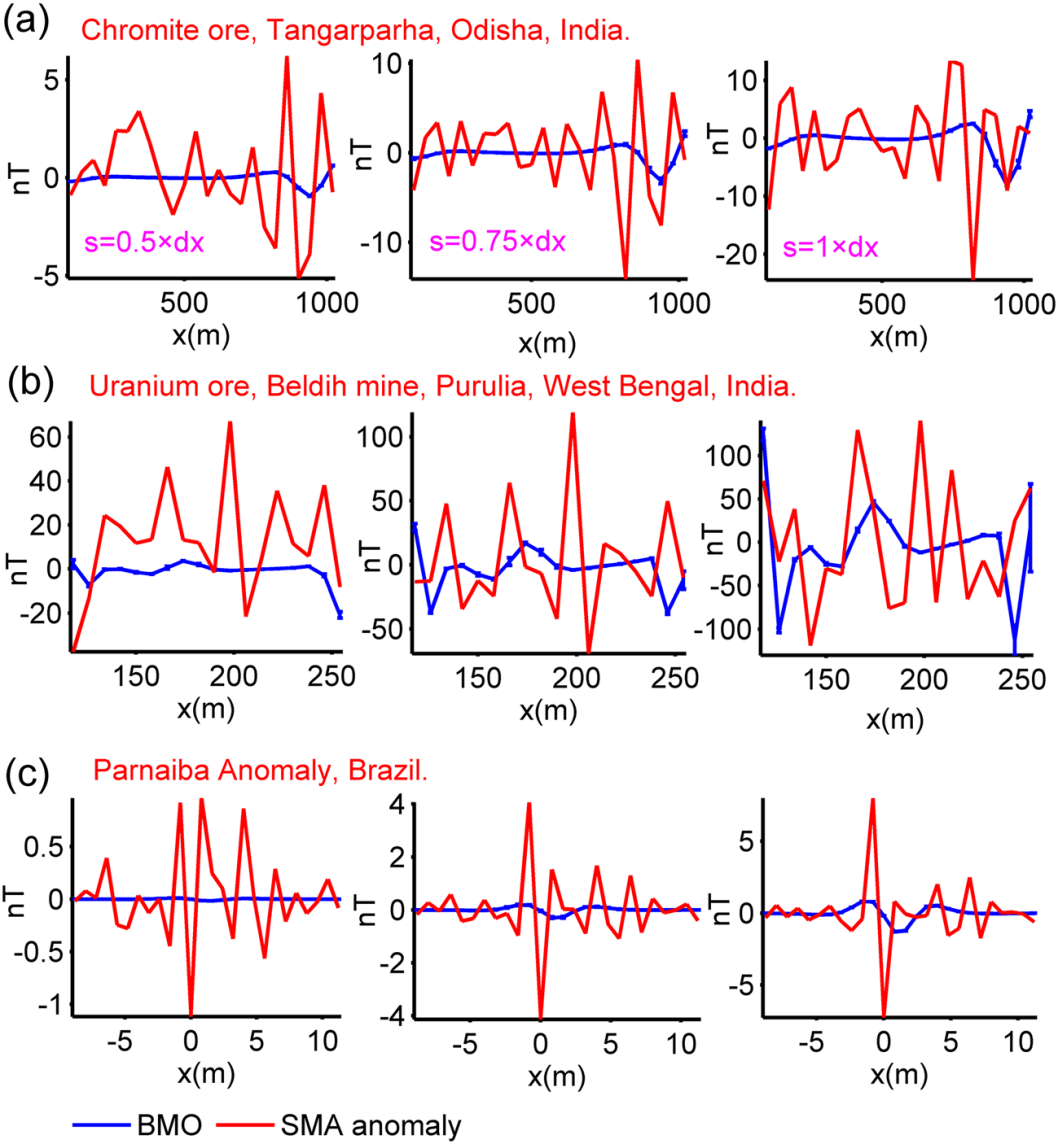
Inversion results of three field cases obtained via BMO with the implementation of the SMA technique, (**a**) the calculated SMA magnetic anomalies of the chromite ore anomaly, India and estimated response of BMO for different *s* values (0.5, 0.75, and 1 × dx, dx = 40 m), (**b**) the calculated SMA magnetic anomalies of the uranium ore anomaly, India and estimated response of BMO for different *s* values (0.5, 0.75, and 1 × dx, dx = 8 m), (**c**) the calculated SMA magnetic anomalies of the Mesozoic dyke anomaly, Brazil and estimated response of BMO for different *s* values (0.5, 0.75, and 1 × dx, dx = 0.8 m).

Figure 16a shows a magnetic anomaly observed on a Mesozoic dyke that intruded into Paleozoic sedimentary rocks in the Parnaiba basin, Brazil^84^. The data sampling interval is 0.8 m. This anomaly was previously interpreted using different approaches^12,26,84,85^ assuming a horizontal cylinder along with thin-sheet structure. Solutions reported in those studies are given in Table 4. Here, this magnetic anomaly was studied using BMO and sPSO algorithms, considering it the response of a cylindrical body. Model parameter search bounds used are listed in Table 5. The run-time of sPSO and BMO were 20 s and 18 s, respectively. Estimated mean responses through BMO and sPSO are displayed in Fig. 16a, which closely match with the measured one (pink circles). However, the unsatisfactory performances of sPSO and BMO are observed at the pointed part shown in Fig. 16a. The possible explanation for this case is the presence of the local interference effect. Figure 16b demonstrates the calculated std values of obtained responses at each measurement station. Figure 16c,d display the mean value and std values of *RMSE* against the iterations. Table 5 gives the estimated model parameters. Findings showed that BMO yields better performance in terms of relatively lower misfit values and higher stability again. Figure 17c shows a comparison between the calculated SMA results for various *s* values (*s* = 0.5, 0.75, and 1 × dx, dx = 0.8 m), and regenerated responses using BMO. The estimated source parameters are listed in Table 4 along with misfits. Accordingly, it can be mentioned that BMO produced steady solutions with those delineated from considering the observed data as a pure residual response. Calculated anomalies of the BMO, however, revealed obvious differences most likely due to the existence of local interference effects and/or random noises. Thus, we concluded that the observed magnetic anomaly does not contain remarkable regional component, but may contain some amount of local perturbation and/or random noise.

### Post-inversion uncertainty appraisal analyses of real data cases

**Figure 18:**
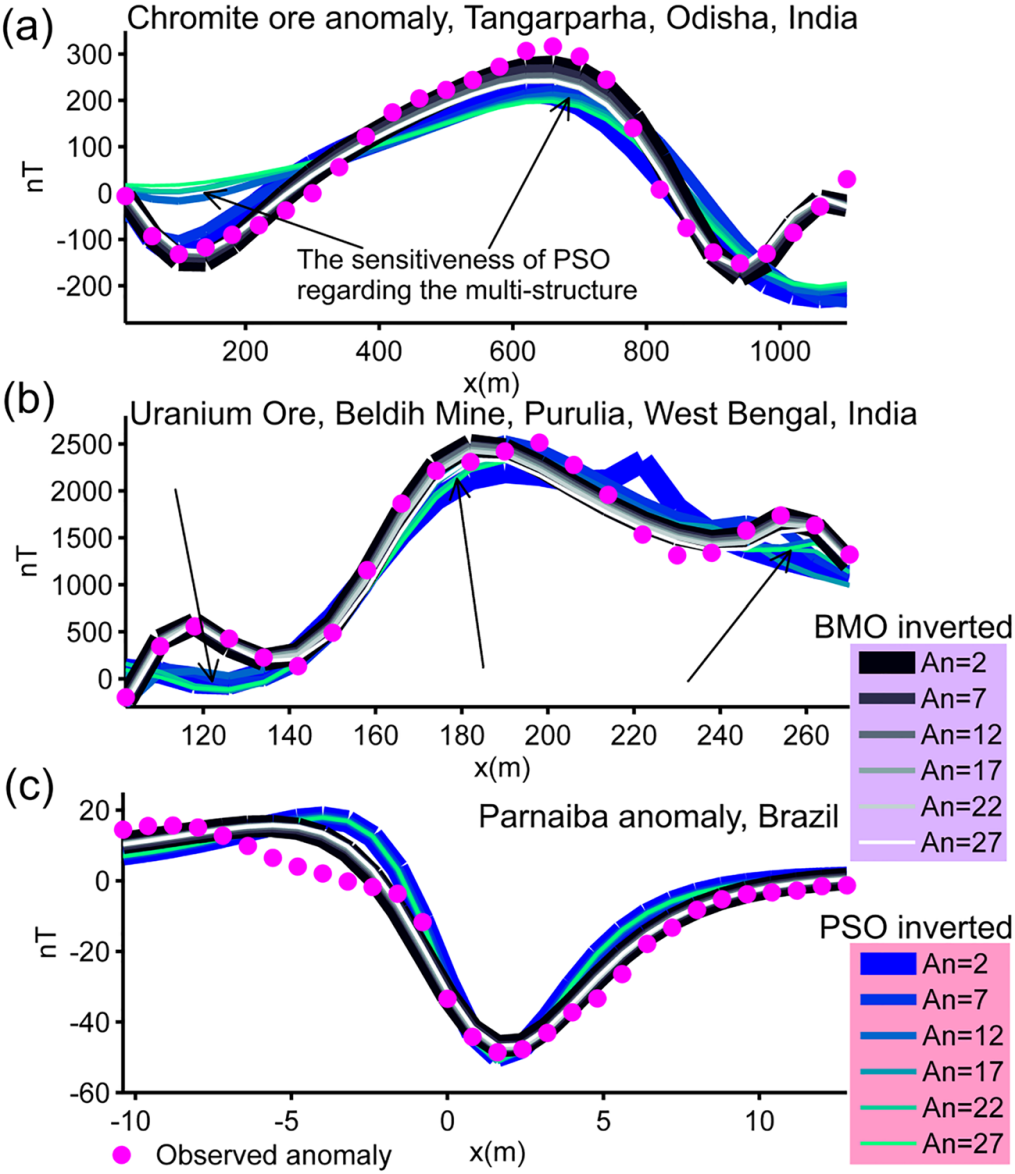
Post-inversion uncertainty appraisal analyses of field data sets using computed mean responses of BMO and sPSO with different *An*. (**a**) chromite ore anomaly, India, (**b**) uranium ore anomaly, India, (**c**) Mesozoic dyke anomaly, Brazil.

The reliability of the model parameters obtained was investigated, as in the synthetic experiments on the basis of the solutions of 30 independent runs for all real data cases. Figure 18 shows the variation characteristics of computed responses using different *An* values. It can be easily observed that the performance of the sPSO was affected with a larger *An* regarding the first two multiple-source model, especially in the pointed parts. This fact correlates with the large std values as discussed before. On the contrary, BMO is generally insensitive to a larger *An*, which indicates the superiority of the BMO in handling more complicated cases. Notably, as for the single structure model, only minor variations occurred in the responses of BMO and sPSO. However, when comparing the best performances of both approaches using *An* = 2 it is again apparent that BMO provided better fitting performance in these real data cases, since it offers sufficient opportunities to escape massive local minima while effectively exploiting the global minimum.

## Conclusions

BMO, a novel nature-inspired derivative-free global optimizer, was presented for the inversion of magnetic anomalies. Modal analyses helped us understand the characteristic of the inverse problem. Finely-tuned control parameter increased the effectiveness of the optimizer. Satisfactory solutions were obtained in both synthetic and real data cases. SMA technique successfully suppressed the regional background effects. The effectiveness of BMO was compared with sPSO, the most widely used metaheuristic in geophysical inverse problems. The reliability of the results produced by both algorithms was tested with uncertainty analysis. Applications showed that BMO produced more stable and more robust solutions for the cases used in this study. It was also observed that BMO completed the optimizations in relatively less run-time than sPSO. Besides, the model parameters obtained for the real data cases are in agreement with the available geologic and/or geophysical information. Based on these findings, we concluded that BMO is more efficient than sPSO in balancing global exploration (the ability to explore the search space extensively and escape the capture of massive local minima) and the local exploitation (intensively approximating the global minimum) processes in this type of inverse magnetic problem. The proposed optimizer quantitatively simulates the specific mating behavior of barnacles leads to a suitable trade-off between these two significant key processes. This study therefore represents an entirely new and competing tool for the efficient interpretation of causative sources from a variety of geophysical anomalies.

On the other hand, similar to other metaheuristic algorithms, one of the weaknesses of the BMO is that it uses an algorithm-based control parameter (*pl*) whose optimum value may change according to each inverse problem. Therefore, parameter tuning studies are needed for each inverse problem in order to get the most out of the algorithm. Another weak point is that it requires more run-time than derivative-based local search algorithms. In addition, the increase in the number of model parameters to be estimated or the studies in which dense anomaly equations are used increase the run-times even more. However, the use of powerful and capable computers produced in line with technological developments can easily reduce this disadvantage.

## Data Availability

The data are available upon request from the first author.

## References

[1] Nykänen V, Raines GL (2006). Quantitative analysis of scale of aeromagnetic data raises questions about geologic-map scale. Nat. Resour. Res..

[2] Abdelrahman EM, El-Araby TM, Soliman KS, Essa KS, Abo-Ezz ER (2007). Least-squares minimization approaches to interpret total magnetic anomalies due to spheres. Pure Appl. Geophys..

[3] Ekinci YL, Yiğitbaş E (2012). A geophysical approach to the igneous rocks in the Biga Peninsula (NW Turkey) based on airborne magnetic anomalies: Geological implications. Geodin. Acta.

[4] Ekinci YL, Balkaya Ç, Şeren A, Kaya MA, Lightfoot C (2014). Geomagnetic and geoelectrical prospection for buried archaeological remains on the upper city of Amorium, a Byzantine city in Midwestern Turkey. J. Geophys. Eng..

[5] Ekinci YL (2016). MATLAB-based algorithm to estimate depths of isolated thin dike-like sources using higher-order horizontal derivatives of magnetic anomalies. Springerplus.

[6] Afshar A, Norouzi GH, Moradzadeh A, Riahi MA (2018). Application of magnetic and gravity methods to the exploration of sodium sulfate deposits, case study: Garmab mine, Semnan, Iran. J. Appl. Geophys..

[7] Essa KS, Nady AG, Mostafa MS, Elhussein M (2018). Implementation of potential field data to depict the structural lineaments of the Sinai Peninsula, Egypt. J. Afr. Earth Sci..

[8] Ekinci YL, Büyüksaraç A, Bektaş Ö, Ertekin C (2020). Geophysical investigation of Mount Nemrut stratovolcano (Bitlis, Eastern Turkey) through aeromagnetic anomaly analyses. Pure Appl. Geophys..

[9] Essa KS, Elhussein M (2020). Interpretation of magnetic data through particle swarm Optimization: Mineral exploration cases studies. Nat. Resour. Res..

[10] Lu N, Liao G, Xi Y, Zheng H, Ben F, Ding Z, Du L (2021). Application of airborne magnetic survey in deep iron ore prospecting—A case study of Jinling area in Shandong Province, China. Minerals.

[11] Essa KS, Munschy M, Youssef MAS, Khalaf EA (2022). Aeromagnetic and radiometric data interpretation to delineate the structural elements and probable precambrian mineralization zones: A case study, Egypt. Min. Metall. Explor..

[12] Biswas A (2018). Inversion of source parameters from magnetic anomalies for mineral/ore deposits exploration using global optimization technique and analysis of uncertainty. Nat. Resour. Res..

[13] Abdelrahman EM, El-Araby TM, Essa KS (2003). A least-squares minimisation approach to depth, index parameter, and amplitude coefficient determination from magnetic anomalies due to thin dykes. Explor. Geophys..

[14] Abdelrahman EM, Abo-Ezz ER, Essa KS (2012). Parametric inversion of residual magnetic anomalies due to simple geometric bodies. Explor. Geophys..

[15] Biswas A (2016). Interpretation of gravity and magnetic anomaly over thin sheet-type structure using very fast simulated annealing global optimization technique. Model. Earth Syst. Environ..

[16] Essa KS, Elhussein M (2019). Magnetic interpretation utilizing a new inverse algorithm for assessing the parameters of the buried inclined dike-like geologic structure. Acta Geophys..

[17] Mehanee S, Essa KS, Diab ZE (2021). Magnetic data interpretation using a new r-parameter imaging method with application to mineral exploration. Nat. Resour. Res..

[18] Gay P (1965). Standard curves for magnetic anomalies over long horizontal cylinders. Geophysics.

[19] McGrath PH, Hood PJ (1970). The dipping dike case: A computer curve matching method of magnetic interpretation. Geophysics.

[20] Nuamah DOB, Dobroka M (2019). Inversion-based Fourier transformation used in processing non-equidistantly measured magnetic data. Acta Geod. Geophys..

[21] Dondurur D, Pamuku OA (2003). Interpretation of magnetic anomalies from dipping dike model using inverse solution, power spectrum and Hilbert transform methods. J. Balk. Geophys. Soc. BGS.

[22] Asfahani J, Tlas M (2007). A robust nonlinear inversion for the interpretation of magnetic anomalies caused by faults, thin dikes and spheres like structure using stochastic algorithms. Pure Appl. Geophys..

[23] Abdelrahman EM, Soliman KS, El-Araby TM, Abo-Ezz ER, Essa KS (2009). A least-squares standard deviation method to interpret magnetic anomalies due to thin dikes. Near Surf. Geophys..

[24] Abo-Ezz ER, Essa KS (2016). A least-squares minimization approach for model parameters estimate by using a new magnetic anomaly formula. Pure Appl. Geophys..

[25] Reid AB, Ebbing J, Webb SJ (2014). Avoidable Euler errors—The use and abuse of Euler deconvolution applied to potential fields. Geophys. Prospect..

[26] Tlas M, Asfahani J (2015). The simplex algorithm for best-estimate of magnetic parameters related to simple geometric-shaped structures. Math. Geosci..

[27] Ma G, Liu C, Xu J, Meng Q (2017). Correlation imaging method based on local wavenumber for interpreting magnetic data. J. Appl. Geophys..

[28] Essa KS, Mehanee S, Elhussein M (2021). Magnetic data profiles interpretation for mineralized buried structures identification applying the variance analysis method. Pure Appl. Geophys..

[29] Abdelrahman EM, El-Araby HM, El-Araby HM, Essa KS (2003). A least-squares minimization approach to depth determination from magnetic data. Pure Appl. Geophys..

[30] Essa KS, Elhussein M (2017). A new approach for the interpretation of magnetic data by a 2-D dipping dike. J. Appl. Geophys..

[31] Kelemework Y, Fedi M, Milano M (2021). A review of spectral analysis of magnetic data for depth estimation. Geophysics.

[32] Ekinci YL (2017). Application of enhanced local wave number technique to the total field magnetic anomalies for computing model parameters of magnetized geological structures. Geol. Bull. Turk..

[33] Zhdanov, M. S. *Geophysical**Inverse**Theory**and**Regularization**Problem* (Elsevier, 2002).

[34] Biswas A, Acharya T (2016). A Very fast simulated annealing (VFSA) method for inversion of magnetic anomaly over semi-infinite vertical rod-type structure. Model. Earth Syst. Environ..

[35] Wang, Y. *Seismic**Inversion:**Theory**and**Applications:**Seismic**Inversion:**Theory**and**Applications* (Wiley, 2016).

[36] Ekinci YL, Balkaya Ç, Göktürkler G, Özyalın Ş (2021). Gravity data inversion for the basement relief delineation through global optimization: A case study from the Aegean Graben System, Western Anatolia, Turkey. Geophys. J. Int..

[37] Ekinci YL, Balkaya Ç, Göktürkler G (2019). Parameter estimations from gravity and magnetic anomalies due to deep-seated faults: Differential evolution versus particle swarm optimization. Turk. J. Earth Sci..

[38] Biswas A, Rao K, Mondal TS (2022). Inverse modeling and uncertainty assessment of magnetic data from 2D thick dipping dyke and application for mineral exploration. J. Appl. Geophys..

[39] Sörensen, K., Sevaux, M. & Glover, F. A history of metaheuristics. In *Handbook**of**Heuristics* (eds. Martí, R., Panos, P., Resende, M.) (Springer, 2018).

[40] Sörensen, K. & Glover, F. W. Metaheuristics. In *Encyclopedia**of**Operations**Research**and**Management**Science*, 663 3rd ed. (eds. Gass, S. I. & Fu, M. C.) 960–970 (2013).

[41] Pace F, Santilano A, Godio A (2021). A review of geophysical modeling based on Particle Swarm Optimization. Surv. Geophys..

[42] Yuan S, Wang S, Tian N (2009). Swarm intelligence optimization and its application in geophysical data inversion. Appl. Geophys..

[43] Fernández Martínez JL, García Gonzalo E, Fernández Álvarez JP, Kuzma HA, Menéndez Pérez CO (2010). PSO: A powerful algorithm to solve geophysical inverse problems. J. Appl. Geophys..

[44] Song X, Tang L, Lv X, Fang H, Gu H (2012). Application of particle swarm optimization to interpret Rayleigh wave dispersion curves. J. Appl. Geophys..

[45] Pace F, Godio A, Santilano A, Comina C (2019). Joint optimization of geophysical data using multi-objective swarm intelligence. Geophys. J. Int..

[46] Abdelrahman EM, Abo-Ezz ER, Soliman KS, El-Araby TM, Essa KS (2007). A least-squares window curves method for interpretation of magnetic anomalies caused by dipping dikes. Pure Appl. Geophys.

[47] Srivastava S, Datta D, Agarwal BNP, Mehta S (2014). Applications of ant colony optimization in determination of source parameters from total gradient of potential fields. Near Surf. Geophys..

[48] Kaftan İ (2017). Interpretation of magnetic anomalies using a genetic algorithm. Acta Geophys..

[49] Ekinci YL, Özyalın Ş, Sındırgı P, Balkaya G, Göktürkler G (2017). Amplitude inversion of 2D analytic signal of magnetic anomalies through differential evolution algorithm. J. Geophys. Eng..

[50] Agarwal A, Chandra A, Shalivahan S, Singh RK (2018). Grey wolf optimizer: A new strategy to invert geophysical data sets. Geophys. Prospect..

[51] Essa KS, Elhussein M (2018). PSO (particle swarm optimization) for interpretation of magnetic anomalies caused by simple geometrical structures. Pure Appl. Geophys..

[52] Di Maio R, Milano L, Piegari E (2020). Modeling of magnetic anomalies generated by simple geological structures through Genetic-Price inversion algorithm. Phys. Earth Planet. Interiors.

[53] Essa KS, Diab ZE (2022). An automatic inversion approach for magnetic data applying the global bat optimization algorithm (GBOA): Application to ore deposits and basement rock intrusion. Geomech. Geophys. Geo-energ. Geo-resour..

[54] Balkaya A, Kaftan I (2021). Inverse modelling via differential search algorithm for interpreting magnetic *anomalies* caused by 2D dyke-shaped bodies. J. Earth Syst. Sci..

[55] Du W, Cheng L, Li Y (2021). l_p_ Norm smooth inversion of magnetic anomaly based on improved Adaptive Differential Evolution. Appl. Sci..

[56] Essa KS, Diab ZE (2022). Magnetic data interpretation for 2D dikes by the metaheuristic bat algorithm: Sustainable development cases. Sci. Rep..

[57] Ben UC, Ekwok SE, Akpan AE, Mbonu CC, Eldosouky AM, Abdelrahman K, Gómez-Ortiz D (2022). Interpretation of magnetic anomalies by simple geometrical structures using the manta-ray foraging optimization. Front. Earth Sci..

[58] Ben UC, Akpan AE, Urang JG, Akaerue EI, Obianwu VI (2022). Novel methodology for the geophysical interpretation of magnetic anomalies due to simple geometrical bodies using social spider optimization (SSO) algorithm. Heliyon.

[59] Sohouli AN, Molhem H, Zare-Dehnavi N (2022). Hybrid PSO-GA algorithm for estimation of magnetic anomaly parameters due to simple geometric structures. Pure Appl. Geophys..

[60] Rao PTKS, Subrahmanyam M (1988). Characteristic curves for inversion of magnetic anomalies of spherical ore bodies. Pure Appl. Geophys..

[61] Rao BSR, Rao TKSP, Murthy ASK (2006). A note on magnetized spheres. Geophys. Prospect..

[62] Rao T, Subrahmanyam M, Murthy AS (1986). Nomogram for the direct interpretation of magnetic anomalies due to long horizontal cylinders. Geophysics.

[63] Gay P (1963). Standard curves for interpretation of magnetic anomalies over long tabular bodies. Geophysics.

[64] Atchuta Rao D, Ram Babu HV, Sankar Narayan PV (1980). Relationship of magnetic anomalies due to surface features and the interpretation of sloping contacts. Geophysics.

[65] Sulaiman MH, Mustaffa Z, Saari MM, Daniyal H (2020). Barnacles Mating Optimizer: A new bio-inspired algorithm for solving engineering optimization problems. Eng. Appl. Artif. Intell..

[66] Zwickl, D. J. Genetic algorithm approaches for the phylogenetic analysis of large biological sequence datasets under the maximum likelihood criterion. PhD Thesis. The University of Texas at Austin, 115 (2006).

[67] Price K, Storn RM, Lampinen JA (2005). Differential Evolution: A Practical Approach to Global Optimization.

[68] Crow JFH (1999). Weinberg and language impediments. Genetics.

[69] Jia H, Sun K (2021). Improved barnacles mating optimizer algorithm for feature selection and support vector machine optimization. Pattern Anal. Appl..

[70] Ekinci YL, Balkaya Ç, Göktürkler G (2021). Backtracking Search Optimization: A novel global optimization algorithm for the inversion of gravity anomalies. Pure Appl. Geophys..

[71] Balkaya Ç, Ekinci YL, Göktürkler G, Turan S (2017). 3D non-linear inversion of magnetic anomalies caused by prismatic bodies using differential evolution algorithm. J. Appl. Geophys..

[72] Deb K, Gupta H (2006). Introducing robustness in multi-objective optimization. Evol. Comput..

[73] Ray, T. Constrained robust optimal design using a multiobjective evolutionary algorithm. In *Proceedings**of**the**2002**IEEE**Congress**on**Evolutionary**Computation*, vol. 1 419–424 (IEEE Press, 2002).

[74] Mirjalili S, Lewis A (2016). Obstacles and difficulties for robust benchmark problems: A novel penalty-based robust optimisation method. Inf. Sci..

[75] Ekinci YL, Balkaya Ç, Göktürkler G, Turan S (2016). Model parameter estimations from residual gravity anomalies due to simple-shaped sources using differential evolution algorithm. J. Appl. Geophys..

[76] Mandal A, Mohanty WK, Sharma SP, Gupta S (2015). Laterite covered mafic-ultramafic rocks: potential target for chromite exploration—A case study from southern part of Tangarparha, Odisha. J. Geol. Soc. India.

[77] Mandal A, Biswas MS (2013). Geophysical anomalies associated with uranium mineralization from Beldih mine, South Purulia Shear Zone, India. J. Geol. Soc. India.

[78] Biswas A, Sharma SP (2014). Integrated geophysical studies to elicit the subsurface structures associated with Uranium mineralization around South Purulia Shear Zone, India: A review. Ore Geol. Rev..

[79] Biswas A, Mandal A, Sharma SP, Mohanty WK (2014). Delineation of subsurface structures using self-potential, gravity, and resistivity surveys from South Purulia Shear Zone, India: Implication to uranium mineralization. Interpretation.

[80] Biswas A, Mandal A, Sharma SP, Mohanty WK (2014). Integrating apparent conductance in resistivity sounding to constrain 2d gravity modeling for subsurface structure associated with uranium mineralization across south Purulia Shear Zone, West Bengal, India. Int. J. Geophys..

[81] Katti, V. J., Sen, J., & Bhatt, A. K. Uranium potentiality of South Purulia Shear Zone, Eastern India Shield. In *Presented**in**Technical**Committee**Meeting**on**Low**Grade**Uranium**Deposits* (IAEA, March 29–31, 2010).

[82] Sharma SP, Biswas A, Mittal S (2014). Delineation of extension of uranium mineralization zone using resistivity and very low frequency electromagnetic surveys around South Purulia Shear Zone, India. J. Geol. Soc. India.

[83] Mandal A, Mohanty WK, Sharma SP, Biswas A, Sen J, Bhatt AK (2015). Geophysical signatures of uranium mineralization and its subsurface validation at Beldih, Purulia District, West Bengal, India: A case study. Geophys. Prospect..

[84] Silva J (1989). Transformation of nonlinear problems into linear ones applied to the magnetic field of a two-dimensional prism. Geophysics.

[85] Abdelrahman EM, Essa KS (2015). A new method for depth and shape determinations from magnetic data. Pure Appl. Geophys..

